# Stakeholder identification and prioritization of barriers to One Health implementation in Ghana’s zoonotic disease surveillance and response system: a sequential mixed-methods study

**DOI:** 10.1186/s12913-026-14819-1

**Published:** 2026-05-29

**Authors:** Joannishka K. Dsani, Ana Maria Perez Arredondo, Sandul Yasobant, Sherry Ama Mawuko Johnson, Walter Bruchhausen

**Affiliations:** 1 One Health Graduate School, Center for Development Research (ZEF), University of Bonn, Genscherallee 3, 53113 Bonn, Germany; 2 Section Global Health, Institute for Hygiene and Public Health (IHPH), University Hospital Bonn, Venusberg Campus 1, Building 66, 53127 Bonn, Germany; 3 Centre for One Health Education, Research and Development (COHERD), Indian Institute of Public Health Gandhinagar (IIPHG), Chiloda Road, Lekawada CRPF P.O, Gandhinagar, Gujarat 382042 India; 4 Department of Public Health Science, Indian Institute of Public Health Gandhinagar (IIPHG), Chiloda Road, Lekawada CRPF P.O, Gandhinagar, Gujarat 382042 India; 5 School of Veterinary Medicine, College of Basic and Applied Sciences, University of Ghana, Legon-Accra, Ghana; 6 German-West African Center for Global Health and Pandemic Prevention (G-WAC), partner site, Bonn, Germany

**Keywords:** One Health, Health system governance, Intersectoral collaboration, Health services research, Zoonotic disease surveillance, Stakeholder engagement, Implementation barriers, Ghana

## Abstract

**Background:**

One Health approaches are vital for zoonotic disease control, yet its effective implementation requires fundamental health system reforms for successful multisectoral coordination. Evidence on operational-level barriers and system requirements is urgently needed. This pre-implementation study examined One Health implementation barriers in Ghana across human, animal, and wildlife health sectors, using structured intersectoral dialogue to prioritise barriers and develop collective solutions for policy development.

**Methods:**

A two-phase mixed-methods study was conducted in Ghana’s three largest metropolitan areas. Phase 1 involved interviews with 101 frontline and national stakeholders, with data analysed using reflexive thematic analysis guided by the Consolidated Framework for Implementation Research (CFIR). Phase 2 convened a participatory workshop (*n* = 16) for individual ranking, collective voting and consensus dialogue to prioritise barriers and formulate policy recommendations. Data were analysed using framework analysis, supported by descriptive statistics, and triangulated across phases.

**Results:**

Ten implementation barriers were identified. Critically, eight (80%) reflected Outer Setting determinants (e.g. policy gaps, system structure, and workforce architecture), while two represented Outer–Inner Setting interactions; no barriers were purely organisational. Initially, sectoral priorities (human: leadership; animal: finance; wildlife: service delivery) converged through dialogue. Participants reframed financial constraints as symptoms of a weak system structure, establishing this as the root barrier. Extreme workforce imbalances (e.g. three wildlife officers nationally) emerged as critical constraints, representing a health service planning failure that makes conventional coordination models operationally infeasible. Participants jointly recommended coordination frameworks with clear mandates and interoperable data systems, subsequently acknowledging workforce gaps as a priority omission.

**Conclusions:**

This study illustrates how participatory cross-sectoral dialogue can shift stakeholder perspectives from sector-specific constraints to systemic governance reform. Findings indicate that sustainable One Health implementation requires addressing both governance fragmentation and workforce architecture across ministerial boundaries, challenging assumptions that organisational capacity alone suffices. These insights directly inform Ghana’s national One Health policy development and offer methodological and empirical guidance for multisectoral health system reforms in low- and middle-income countries facing similar implementation challenges.

**Supplementary Information:**

The online version contains supplementary material available at 10.1186/s12913-026-14819-1.

## Background

Zoonotic diseases remain a significant threat to global health security, with recent epidemics and pandemics highlighting the urgent need for integrated approaches to prevention, surveillance, and response [1–3]. The One Health (OH) approach offers a systemic framework that recognises the interdependence of human, animal, and environmental health [4]. As most emerging infectious diseases originate in animals before spilling over into human populations, zoonotic disease surveillance and response (ZDSR) sits at the core of OH implementation [4, 5]. Effective control requires cross-sectoral collaboration to detect, monitor, and respond to outbreaks at their source, often within animal populations or shared ecosystems [4–6].

Despite OH gaining global momentum and endorsement from major health bodies, countries often face difficulties translating these principles into routine practice [7–9]. OH programmes and initiatives, such as joint zoonotic disease surveillance across human and animal health systems, coordinated outbreak investigation, and shared data reporting platforms, require sustained cross-sectoral collaboration to function effectively.

Barriers to such collaboration can arise at two distinct stages: prior to policy enactment, where challenges relate to defining institutional mandates, securing political commitment, and establishing governance frameworks; and after policy enactment, where operational barriers emerge in implementing programmes within and across existing health systems [10, 11].

Across diverse health system contexts, OH efforts are often hindered by fragmented governance, sectoral silos, and unclear mandates. These challenges have been documented in both resource-limited and high-income settings [5, 8, 9, 12, 13]. In low- and middle-income countries (LMICs), however, they are often compounded by limited resources, weaker institutional infrastructure, and competing health priorities, making the implementation gap particularly acute [5, 8, 9].

However, context-specific evidence on how these barriers are perceived and prioritised by frontline stakeholders in particular country settings remains limited. Understanding these operational-level perspectives is critical for designing effective, context-sensitive implementation strategies [5, 8].

One Health operationalisation presents a fundamental health services research challenge: unlike reforms that modify practices within a single sector, it requires structural changes to how health systems are governed, how surveillance systems are organised across organisational boundaries, and how health workforces collaborate across traditionally separate sectors for effective disease surveillance and response [14–17]. Yet the specific barriers constraining OH implementation for ZDSR remain poorly understood in many LMIC contexts. Implementation science frameworks increasingly recognise that translating evidence into practice requires understanding not only technical capacity but the governance, policy, and organisational contexts that enable or constrain implementation [18–21]. By investigating One Health implementation through a health systems lens, this study contributes evidence relevant both to zoonotic disease preparedness and to broader health service delivery reform requiring sustained intersectoral coordination.

Ghana, a country with a history of zoonotic outbreaks such as anthrax, rabies, and avian influenza, joined the Global Health Security Agenda in 2015 to promote OH initiatives and strengthen its capacity for multisectoral disease response [22–25]. National efforts have since included the formation of a One Health Technical Working Group (OHTWG) and the development of a draft OH policy, which is still under review [22, 26, 27].

Ghana’s ZDSR system is organised across three institutional sectors operating under separate ministerial frameworks. Human health surveillance is the responsibility of the Disease Surveillance Department within the Public Health Division of the Ghana Health Service (GHS), which operates under the Ministry of Health through a hierarchical national-regional-district structure established under the Ghana Health Service and Teaching Hospitals Act, 1996 (Act 525) [28]. Policy authority is determined at the national level and implemented through regional and district directorates, with district directors accountable upward through regional directors to the Director-General [28]. Animal health surveillance is managed by the Epidemiology Unit of the Veterinary Services Department (VSD), operating under the Ministry of Food and Agriculture, with district veterinary officers deployed across district capitals [29]. The Wildlife Division (WD) of the Ghana Forestry Commission, established under the Forestry Commission Act, 1999 (Act 571), bears responsibility for wildlife health [30]. In the absence of a dedicated wildlife disease surveillance framework, wildlife health issues including zoonotic disease are managed in accordance with VSD policies [31]. This centralized, sector-specific governance architecture, in which each sector operates under a distinct ministry with its own hierarchical reporting structure, creates the inter-organisational and policy fragmentation that makes multisectoral OH coordination structurally challenging even before any formal implementation begins. As Ghana advances its national OH implementation plan, empirical evidence on implementation barriers is urgently needed to inform policy development and resource allocation decisions [5, 8, 32].

Despite growing interest in One Health, much of the existing literature remains conceptual or limited to specific disease case studies, with relatively few empirical studies investigating implementation barriers from the perspective of frontline stakeholders [6, 8, 33–37]. Yet these perspectives are critical, since successful implementation depends on how stakeholders, particularly those working closest to zoonotic disease events, perceive and navigate multisectoral collaboration challenges [38–40]. In Ghana, where OH policy and institutional arrangements are still under development [27, 41], this evidence gap is particularly pressing. To address it, this study applies the Consolidated Framework for Implementation Research (CFIR) to systematically identify and categorise multilevel barriers influencing ZDSR.

CFIR is a comprehensive determinant framework that synthesizes constructs from multiple implementation theories and organises them into five major domains: Innovation (the intervention), Outer Setting (external environment), Inner Setting (internal context), Individuals (involved actors), and Process (implementation strategies) [11, 42]. The Outer Setting encompasses broader structural and policy environments, including governance arrangements, financing mechanisms, and inter-organisational relationships, while the Inner Setting refers to organisational-level features such as institutional culture, leadership structures, and resource availability. Because OH operationalisation requires coordination across multiple sectors and institutions, CFIR offers a particularly suitable lens for examining how governance structures, policy environments, and organisational arrangements shape the feasibility of multisectoral collaboration. This is especially relevant in Ghana’s context, where OH governance structures are still being formalised. Consistent with CFIR’s prospective assessment approach, designed for use before adoption and implementation occurs, this study examines multilevel determinants of anticipated implementation outcomes at this policy formalisation stage [10, 43].

Specifically, this study aimed to (1) identify perceived barriers to OH operationalisation from frontline stakeholder perspectives, (2) assess how these barriers were prioritised across sectors, and (3) document collaboratively proposed solutions. In doing so, this study generates policy-relevant evidence to inform Ghana’s ongoing OH governance and coordination efforts and offers insights for strengthening health system structures to address complex multisectoral challenges, particularly in LMIC settings facing similar institutional constraints.

## Methods

### Study design and setting

This mixed-methods study employed a sequential exploratory design to explore stakeholder perspectives on OH implementation barriers in Ghana’s ZDSR systems. Phase 1 involved qualitative in-depth interviews to identify implementation barriers, and Phase 2 comprised a participatory prioritization workshop to rank these barriers and co-develop solutions. This approach enabled comprehensive understanding of implementation barriers and stakeholder-informed implementation strategies.

Data were collected across four strategic locations representing Ghana’s geographic and administrative diversity: Greater Accra Metropolitan Area (GAMA, southern belt), Kumasi Metropolitan Area (KMA, middle belt), Tamale Metropolitan Area (TAMA, northern belt), and national-level offices in Accra, which allowed us to capture variations in the Inner and Outer Settings of the implementation context.

This study was part of a larger research project examining OH implementation in Ghana’s ZDSR system across multiple objectives, including an evaluation of existing intersectoral collaboration practices [31, 44], an assessment of individual sectoral surveillance systems, and an investigation of facilitators and opportunities for OH implementation. The present paper focuses specifically on stakeholder perspectives on implementation barriers.

### Participants and sampling

**Study population:** Participants were drawn from three sectors: Ghana Health Service representing human health (HH), Veterinary Services Department representing domestic animal health (AH), and Wildlife Division (WD) representing wildlife health (WH).

Although environmental health institutions were considered during the project design phase, a stakeholder mapping exercise conducted prior to data collection, comprising a workshop and individual consultations with key institutional representatives, found that neither the Environmental Health Department under the Ministry of Local Government and Rural Development nor the Environmental Protection Unit within the Ministry of Environment, Science, Technology, and Innovation (MESTI) had established zoonotic disease surveillance systems. The larger research programme from which this paper is drawn therefore focused on the three sectors with active ZDSR systems.

**Sampling strategy:** Purposive sampling primarily targeted frontline implementers at district and sub-district levels, who serve as key actors and a primary source of information on the Inner Setting (e.g., organisational practices, resources, and culture). This was supplemented by national and regional staff to provide a multi-level perspective on the Outer Setting (e.g., policy and inter-organisational relations). Inclusion criteria: (1) current employment in ZDSR activities, (2) minimum one year of sectoral experience, and (3) willingness to participate.

**Sampling approach:** For the AH and WH sectors, where the ZDSR workforce is small and clearly bounded, sampling aimed to achieve complete or near-complete coverage of available personnel. In contrast, for the HH sector, where ZDSR responsibilities are distributed across a larger and more diverse workforce, purposive sampling targeted personnel with direct ZDSR roles rather than aiming for thematic saturation:

- **Human health:** District health directors and senior disease control/public health officers across study districts, supplemented by additional officers when recommended.
- **Animal health:** District veterinary officers and supporting staff, representing approximately 80-90% of available personnel in the study districts. We interviewed complete district teams in TAMA and KMA, and either complete teams or senior personnel in GAMA districts.
- **Wildlife health:** Unlike the HH and AH sectors, WH officers in Ghana are not based at the district level. We interviewed all personnel responsible for wildlife ZDSR nationwide.

**Geographic sampling:** Within GAMA’s 25 districts, we initially identified 21 districts with both HH and AH personnel. Data collection continued until thematic saturation at 16 districts (76% of eligible districts). KMA and TAMA were sampled as single-district metropolitan areas.

Contact information was obtained from national/regional authorities. No participants refused participation, although some nominated colleagues due to scheduling conflicts.

### Data collection

#### Phase 1: stakeholder interviews (February–May 2023)

Sixty-four interviews were conducted with 101 stakeholders: 41 interviews from HH (*n* = 67 participants), 21 from AH (*n* = 31 participants), and two from WH (*n* = 3 participants). Participant distribution included 83 district-level frontline implementers, 15 regional/national-level staff, and three WH actors. Phase 1 interviews were conducted individually or in a group. Of the 64 interview sessions, 41 were conducted individually and 23 involved small groups of two to four participants. Group participants were from the same sector, the same facility, and the same district, and it was entirely the participants’ choice whether to be interviewed alone or with their working colleagues. These were not focus group discussions, group sessions followed the same semi-structured format as individual interviews. The key question was posed conversationally to the group and then to each participant in turn to ensure all individual opinions were captured. Because participants in group sessions were already colleagues working within the same unit, for example, a veterinary officer and their technicians, or a district director of health and their surveillance officers, the social dynamics differed from those of unfamiliar groups. Participants spoke freely, either affirming previously raised challenges or contributing distinct perspectives. No cross-sector groupings occurred in Phase 1; intersectoral groups were convened only in the Phase 2 workshop.

**Interview procedures:** Semi-structured interviews (20–90 minutes) were conducted using a multi-topic interview guide developed to explore intersectoral collaboration and OH implementation topics comprehensively. The guide was informed by the WHO’s *Surveillance and Response Systems framework* and the Evaluation of collaboration in a multisectoral surveillance system tool, and a portion of it was described in a prior publication [36, 45, 46].

For the present analysis, data were extracted from responses to a key open-ended question that was asked flexibly. The intent was to identify stakeholders’ perceptions of systemic weaknesses that could become implementation barriers. The exact phrasing varied based on the conversational flow, including questions such as, “What do you think is a weakness of the current surveillance system that, if not addressed, would prevent successful OH implementation?” and “What challenges in the system do we need to fix to make this OH thing work?” The core objective remained consistent: to elicit stakeholders’ perceptions of critical implementation barriers.

**Interview settings:** Primarily workplace offices (district directorates, health facilities, veterinary clinics, laboratories). Four interviews were conducted remotely (Zoom/telephone) for accessibility.

#### Phase 2: prioritization workshop (April 2024)

A one-day workshop was conducted in Accra with 16 stakeholders: 7 HH, 7 AH, and 2 WH representatives. Participants included GAMA-based HH and AH actors, plus regional/national-level WH representatives. Ten participants had participated in Phase 1 for continuity. Selection criteria included sectoral expertise, leadership roles, and availability for the workshop.

**Workshop structure:** Six structured activities were facilitated by the research team:

- **Phase 1 findings presentation:** Participants were briefed on the key findings from Phase 1
- **Activity 1 – Individual Ranking**: Participants individually ranked their top 10 implementation barriers (sub-themes) from 35 specific barriers identified in Phase 1. Each barrier included descriptive examples for clarity.
- **Activity 2 - Overarching Thematic Voting:** Ten overarching thematic barriers (derived from Phase 1 analysis) were displayed as posters with detailed descriptions. Participants voted for their top four themes using assigned sector-specific identifiers.
- **Activity 3 - Intersectoral Dialogue:** Voting results were presented and discussed, with participants reflecting on sectoral differences and responding to facilitated prompts about priority differences.
- **Activity 4 - Recommendation Development**: Participants worked in two mixed-sector groups to co-develop actionable recommendations for the top priority implementation barriers identified in preceding activities. Groups were purposively composed to ensure balanced representation across sectors, professional cadres, and levels of the health system.

Group 1 included a wildlife veterinarian, two municipal directors of health, a facility- level medical officer, a municipal disease control officer, two municipal veterinary doctors, and a veterinary technician Group 2 comprised a wildlife veterinarian, a municipal director of health, two disease control officers (municipal and sub-municipal levels), three municipal veterinary doctors, and a veterinary technician

(also serving as a municipal veterinary officer).

Efforts were made to distribute participants such that no single sector or professional cadre dominated group discussions. Both groups received identical instructions: to develop concrete, actionable recommendations addressing the prioritised barriers that could realistically be implemented within Ghana’s existing health system architecture.

- **Plenary Discussion:** Groups presented recommendations, followed by peer feedback and refinement of proposed solutions.

No incentives were provided to Phase 1 participants. Phase 2 workshop participants received lunch and transportation reimbursement in recognition of their time commitment to the full-day event. Both phases were audio-recorded with participant consent. Phase 2 facilitator notes and flipchart outputs were collected. Data quality was ensured through systematic verification of audio recordings, standardised transcription protocols, and field notes capturing contextual observations and non-verbal cues.

### Data analysis

**Qualitative analysis:** Phase one interview transcripts underwent reflexive thematic analysis [47] using MAXQDA software [48], following Braun and Clarke’s six-phase approach (familiarization, coding, theme development, review, refinement, reporting). Themes were primarily derived inductively from the data generating a set of specific barriers that were subsequently consolidated into broader overarching themes. Since the objective of Phase 1, was to identify the full range of implementation challenges perceived across the system, any challenge raised by any participant in a session was documented and consolidated thematically. CFIR’s Inner and Outer Setting domains were then applied to organise and interpret these themes, linking stakeholder-identified barriers to established multilevel implementation determinants. This approach is consistent with established CFIR practice, whereby open data collection techniques are used and CFIR is applied during the analysis and interpretation phases rather than to design data collection instruments [10, 49]. The lead author conducted the primary analysis, and to enhance rigor, 10% of transcripts were independently coded by the third author. Discrepancies were resolved through discussion, and coding was refined through consensus. For Phase 2 discussions, previously identified themes were applied deductively using Framework Analysis [50] to compare sectoral perspectives and track evolving priorities through structured consensus-building.

**Quantitative analysis:** Individual rankings used weighted scoring (Rank 1 = 10 points, Rank 2 = 9 points, through to Rank 10 = 1 point, with unranked barriers receiving 0 points) to create composite priority scores. Sector-specific rankings and combined weighted scores were computed separately to allow both individual sectoral priorities and collective prioritization to be examined. Descriptive statistics included means, medians, and standard deviations. Only barriers ranked by ≥ 3 participants (≥20%) were included. Thematic voting was analysed by overall and sector-specific frequencies.

**Integration:** Triangulation of qualitative dialogue with quantitative rankings enhanced the understanding of both the content and relative priority of implementation barriers, revealing how structured dialogue influenced stakeholder perspectives.

## Methodological rigor

Study rigor was enhanced through prolonged engagement across multiple phases, triangulation of methods and data sources, reflexive journaling, and peer review of analytical interpretations. Member checking was conducted through a comprehensive presentation of Phase 1 findings to Phase 2 participants at the workshop opening, providing them with opportunities to comment, validate, or suggest modifications to preliminary results before proceeding with prioritization activities. This study follows the *Consolidated Criteria for Reporting Qualitative Research* (COREQ) guidelines.

### Researcher positionality and reflexivity

The Phase one qualitative interviews were conducted by the first author (J.D.), a Ghanaian Doctor of Veterinary Medicine holder and Public Health PhD candidate with prior experience in zoonotic disease surveillance in Ghana. The Phase two workshop was co-facilitated by J.D. and the second author (A.P.), a PhD candidate in Development Research focusing on institutional change. Both researchers had a deep contextual understanding of One Health in Ghana.

J. D’s veterinary background and professional experience within the system necessitated reflexive attention to potential Animal Health and Wildlife Health biases. This insider familiarity enabled recognition of subtle interpersonal dynamics during group sessions, including whether participants were speaking freely or deferring to colleagues, which informed real-time probing to encourage the expression of individual perspectives.

Across both individual and group settings, participants appeared generally comfortable sharing their views and engaged openly in discussion. Reflexivity was maintained through systematic memo-writing after each interview documenting researcher assumptions and reactions, peer debriefing sessions with co-authors from different disciplinary backgrounds, explicit acknowledgment of potential professional bias in analytical discussions, and regular reflection on how insider knowledge might influence data interpretation to enhance analytical objectivity.

## Results

### Phase 1 results: identifying implementation barriers

Phase 1 results present the identified themes and their explanations without sectoral or demographic breakdowns, reflecting the study’s aim at this stage to map the landscape of challenges rather than compare perspectives across groups.

Analysis of the interviews identified 10 overarching implementation barrier themes, which were systematically categorised using the Inner and Outer Setting domains of the CFIR. The framework revealed that eight of these themes were situated in the Outer Setting, whereas two represented a complex interplay between the Outer and Inner Setting domains. These themes encompassing 35 specific barriers were consistent across sectors and regions. However, closer examination revealed contextual nuances that became clearer during Phase 2. The themes and their key characteristics are presented in Table 1, with supporting quotations in the Appendix (Additional file 1). To protect participant confidentiality due to the sensitive nature of the study and small sector sizes, detailed demographic data (such as gender, years of experience, district name, and professional cadre) are not reported. Instead, the sample is described broadly by sector (HH, AH, WH).

**Table 1:** Overarching implementation barriers to One Health in Ghana’s zoonotic disease surveillance and response system, organised by the Consolidated Framework for Implementation Research (CFIR) domains

CFIR Domains	Theme	Key Challenges	Description
Outer + Inner Setting	Financial Challenges	Funding shortfalls, mismanagement, corruption, unsustainable financing	Persistent funding shortfalls, particularly for surveillance, infrastructure, and emergency response, would significantly hinder OH implementation. Mismanagement, corruption, and unsustainable financing mechanisms were flagged as systemic weaknesses likely to undermine coordination and operations.
Outer Setting	System Structure Challenges	Sectoral misalignments, siloed governance, distinct hierarchies, unharmonized reporting	Structural misalignments across sectors create major barriers with siloed governance systems having distinct hierarchies, reporting channels, and surveillance structures lacking intersectoral collaboration mechanisms. Without harmonized reporting tools, geographic zones, and technical language, OH efforts would remain fragmented.
Outer Setting	Data sharing and communication challenges	Siloed data systems, sector-specific reporting, absence of joint platforms	Siloed data systems, sector-specific reporting mechanisms, and the absence of joint platforms or standardized communication channels were viewed as critical barriers to timely coordination. Limited visibility into other sectors’ data weakened situational awareness, mutual accountability, and outbreak response.
Outer Setting	Lack of Policies and Legislation	Unclear policies, absent legal frameworks, vague mandates	The absence of clear policies and legal frameworks was anticipated to undermine OH institutionalization, especially at subnational levels. Vague mandates and undefined sectoral roles enabled overlap, confusion, and intersectoral conflict.
Outer Setting	Lack of Leadership on Collaboration	No designated authority, weak enforcement mechanisms, fragmented initiatives	Participants stressed the lack of a designated authority to lead OH coordination. Without formal leadership and enforcement mechanisms, even well-defined policies were seen as unenforceable, resulting in fragmented, short-lived initiatives.
Outer Setting	Lack of Political Will	Weak political commitment, disengaged assemblies, inconsistent prioritization	Weak political commitment was evidenced by disengaged district assemblies, weak enforcement of existing mandates, and inconsistent political prioritization. Political neglect of AH concerns and poor follow-through on mandates reflected this inconsistency.
Outer Setting	Limited Public Awareness	Low community awareness, educational gaps, cultural attitudes	Low community awareness of zoonotic risks and OH principles was considered a serious barrier. Gaps in education, cultural attitudes toward animals, and the absence of clear reporting pathways for the public undermined engagement and behavior change.
Outer Setting	Workforce Gaps and Capacity Limitations	Severe staffing shortages, limited frontline capacity, need for cross-training	Severe staffing shortages, especially in AH and WH sectors and limited frontline capacity were seen as major constraints. Cross-training and structured collaboration among rangers, veterinary staff, and other actors were needed to enhance surveillance and response.
Outer Setting	Service Delivery Difficulties	Limited vaccine access, weak referral systems, fragmented responsibilities	Access to vaccines and essential services was limited by cost, weak referral systems, and fragmented responsibilities. Safety concerns around unregulated distribution and poor oversight were particular issues in rural areas.
Outer + Inner Setting	Low Cross-Sectoral Motivation and Engagement	Low motivation, weak institutional buy-in, passive attitudes	Low motivation and weak institutional buy-in limited implementation. Passive attitudes, dormant platforms, and misaligned sectoral goals reflected deeper attitudinal barriers to intersectoral trust and collaboration.

Legend: This table presents 10 overarching themes identified from interviews with stakeholders in the human, animal, and wildlife health sectors. Each theme is mapped to the corresponding CFIR domain and includes key challenges and detailed descriptions. The mapping illustrates how identified barriers are distributed across system-level (Outer Setting) and organizational-level (Inner Setting) determinants within Ghana’s zoonotic disease surveillance and response system

### Phase 2 results: prioritization workshop

#### Results from workshop Activity 1: individual ranking of 35 specific implementation barriers (sub-themes)

Of the 35 specific implementation barriers identified, participants ranked their top three as: *(1) lack of funds/logistics for surveillance activities* (score: 96), *(2) lack of infrastructure* (score: 68), and (3) *system structure not including collaboration* (score: 67). All three top-ranked barriers reflect Outer Setting constraints beyond frontline implementer control. The full list of 35 specific barriers presented to participants in Phase 2, along with their raw rankings, is included in Additional file 2.

Implementation barrier priorities were largely consistent across sectors, though some differences emerged: WH participants prioritised *inactive surveillance programmes*, HH participants ranked *system structure not including collaboration* highest, and AH participants emphasised *lack of infrastructure*. A comparative breakdown by sector is provided in Table 2. Given the unequal sectoral representation in the workshop (7 HH, 7 AH, 2 WH participants), combined weighted scores reflect predominantly HH and AH perspectives, with WH contributing proportionally less. Table 2 is therefore important for identifying where WH priorities diverged from the overall ranking.

**Table 2:** Sector-specific ranking of the top 10 specific implementation barriers to One Health in Ghana’s zoonotic disease surveillance and response system (Workshop Activity 1: individual ranking)

Rank	Human Health	Animal Health	Wildlife Health
1	Health System structure not including collaboration	Lack of Infrastructure	Inactive Surveillance programs
2	Lack of funds/logistics for surveillance activities	Lack of funds/logistics for surveillance activities	Unsustainable funding sources
			Lack of funds/logistics for surveillance activities
			Corruption
3	Lack of OH Policies and Legislation - General	Lack of funds/logistics for response/control activities	-
4	Unsustainable funding sources	Lack of Knowledge (actors) - Knowledge of zoonosis. Public Health knowledge, Understanding of OH	-
5	Poor implementation strategies	Lack of Political will	Health System structure not including collaboration
6	No OH Leadership	Unsustainable funding sources	Weak technical capacity of actors
7	Differing reporting/data sharing platforms between sectors	Inadequate and/or a lack of staff	Inadequate and/or a lack of staff
		Relationship Issues	Lack of Infrastructure
8	Weak technical capacity of actors	-	-
9	Lack of funds/logistics for response/control activities	Health System structure not including collaboration	High turnover of actors
10	Low visibility and impact of actors	Low visibility and impact of actors	Unmotivated Community Volunteer staff
		Inactive Surveillance Programs	Bureuacracy

Legend: This table presents the results of Workshop Activity 1, in which participants from the human health, animal health, and wildlife health sectors individually ranked their top 10 specific implementation barriers (sub-themes). The table shows sector-specific rankings; in some cases, barriers received the same ranking due to ties

To supplement rankings, a frequency analysis revealed how many participants included each implementation barrier in their top 10. Barriers selected by 10 or more participants indicate widespread agreement (e.g., the top-ranked barrier was chosen by 13), whereas those selected by only one or two suggest more sector-specific concerns (e.g., *OH actors missing from health committees* and *lack of assertiveness*).

Descriptive statistics revealed the lowest mean ranks for *lack of infrastructure* (2.50), *system structure not including collaboration* (2.63), and *lack of funds/logistics for surveillance activities* (3.23), indicating that these themes were seen as most critical. The standard deviations ranged from 1.09 to 3.49, with lower values reflecting greater consensus. Notably, *system structure not including collaboration* had both a low mean and a low standard deviation, suggesting that it was both important and widely agreed upon as an Outer Setting determinant. Full statistics are presented in Table 3.

**Table 3:** Ranking and descriptive statistics of specific implementation barriers to One Health in Ghana’s zoonotic disease surveillance and response system (Workshop Activity 1)

	Challenge	Mean Rank	Standard Deviation	Total Weighted Score	Frequency (n)	% of Participants	Weighted Score Ranking
1	Lack of Infrastructure	2.50	1.94	68	8	50.00	**2nd**
2	Unsustainable funding sources	4.56	2.50	58	9	56.25	**5th**
3	Lack of funds/logistics for surveillance activities	3.23	2.26	96	13	81.25	**1st**
4	Lack of funds/logistics for response/control activities	4.25	1.48	59	8	50.00	**4th**
5	Difficulty Generating Revenue	No Data	No Data	0	0	0.00	35th
6	Corruption	5.25	3.49	23	4	25.00	16th
7	Health System structure not mandating collaboration	2.63	0.99	67	8	50.00	**3rd**
8	OH actors missing from Public (Health) Committees	3.00	0.00	8	1	6.25	26th
9	Inactive Surveillance programs	3.75	1.48	29	4	25.00	11th
10	Relationship Issues	4.00	2.16	21	3	18.75	18th
11	Bureuacracy	7.00	1.00	8	2	12.50	26th
12	Inadequate and/or lack of staff	4.83	2.11	37	6	37.50	8th
13	High turnover of actors	7.50	2.50	7	2	12.50	29th
14	Low visibility and impact of actors	6.17	2.19	29	6	37.50	11th
15	Unmotivated Community Volunteer staff	7.00	1.00	8	2	12.50	26th
16	Weak technical capacity of actors	5.33	1.89	34	6	37.50	9th
17	Lack of Knowledge (actors)(1.knowledge in zoonosis.2. Public Health knowledge 3.Understanding of OH	4.17	3.48	41	6	37.50	7th
18	Lack of Knowledge (Community)	8.00	2.83	9	3	18.75	24th
19	Lack of OH Policies and Legislations - General	5.50	3.16	44	8	50.00	6th
20	Unclear Sector Roles	5.80	2.86	26	5	31.25	15th
21	Lack of Political will	6.14	2.90	34	7	43.75	9th
22	No OH Leadership	4.25	2.49	27	4	25.00	14th
23	Poor implementation strategies	6.17	2.48	29	6	37.50	11th
24	Poor data storage/management	8.50	0.50	5	2	12.50	31st
25	No uniformed data collection protocols	7.20	1.17	19	5	31.25	19th
26	No joint communication platforms	8.40	1.36	13	5	31.25	20th
27	Differing reporting/data sharing platforms between sectors	7.17	1.67	23	6	37.50	16th
28	Difficulty with accessing & availability of other sector’s data	8.25	1.09	11	4	25.00	21st
29	Other sectors’ not open to data sharing	7.50	1.50	7	2	12.50	29th
30	Poor data sharing practices	9.50	0.50	3	2	12.50	34th
31	Lack of assertiveness of actors	7.00	0.00	4	1	6.25	33rd
32	Inferiority complex	8.50	1.50	5	2	12.50	31st
33	Each system has a different objective	8.50	1.12	10	4	25.00	22nd
34	Low interest in collaborating	8.00	1.63	9	3	18.75	24th
35	Difficulty getting needed drugs and vaccines	7.67	1.70	10	3	18.75	22nd

Legend: This table presents descriptive statistics for Workshop Activity 1, in which participants from the human, animal and wildlife health sectors ranked their top 10 specific implementation barriers (sub-themes) to One Health implementation. The top five barriers based on weighted scores are highlighted in bold

### Results from workshop Activity 2: overarching thematic voting

**Fig. 1:**
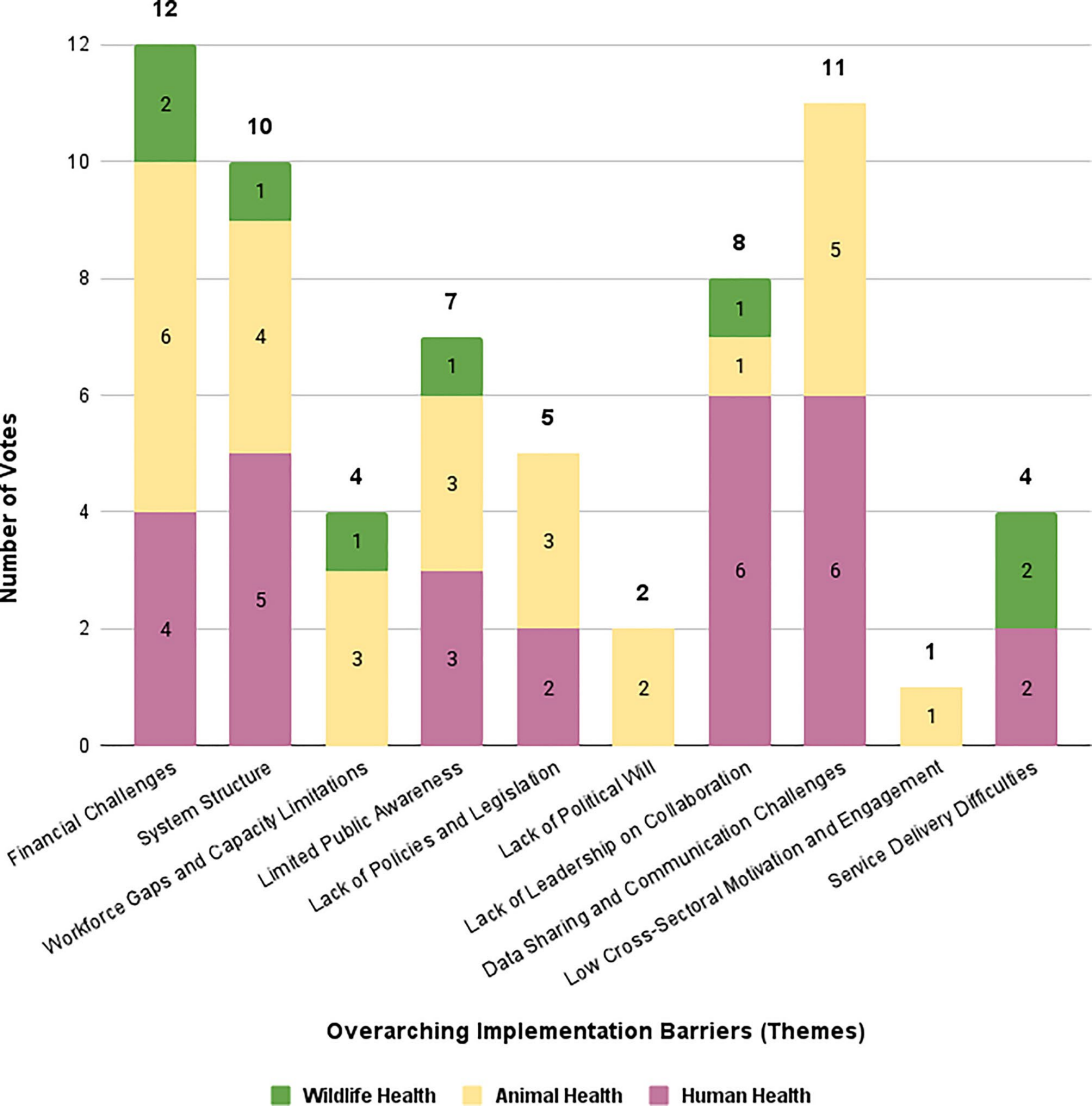
Total votes for each overarching thematic barrier across sectors. Legend: This figure displays the total number of votes each of the ten overarching thematic barriers received during the workshop prioritization exercise (Activity 2). Participants voted for their top four most pressing barriers using sector-specific identifiers

**Fig. 2:**
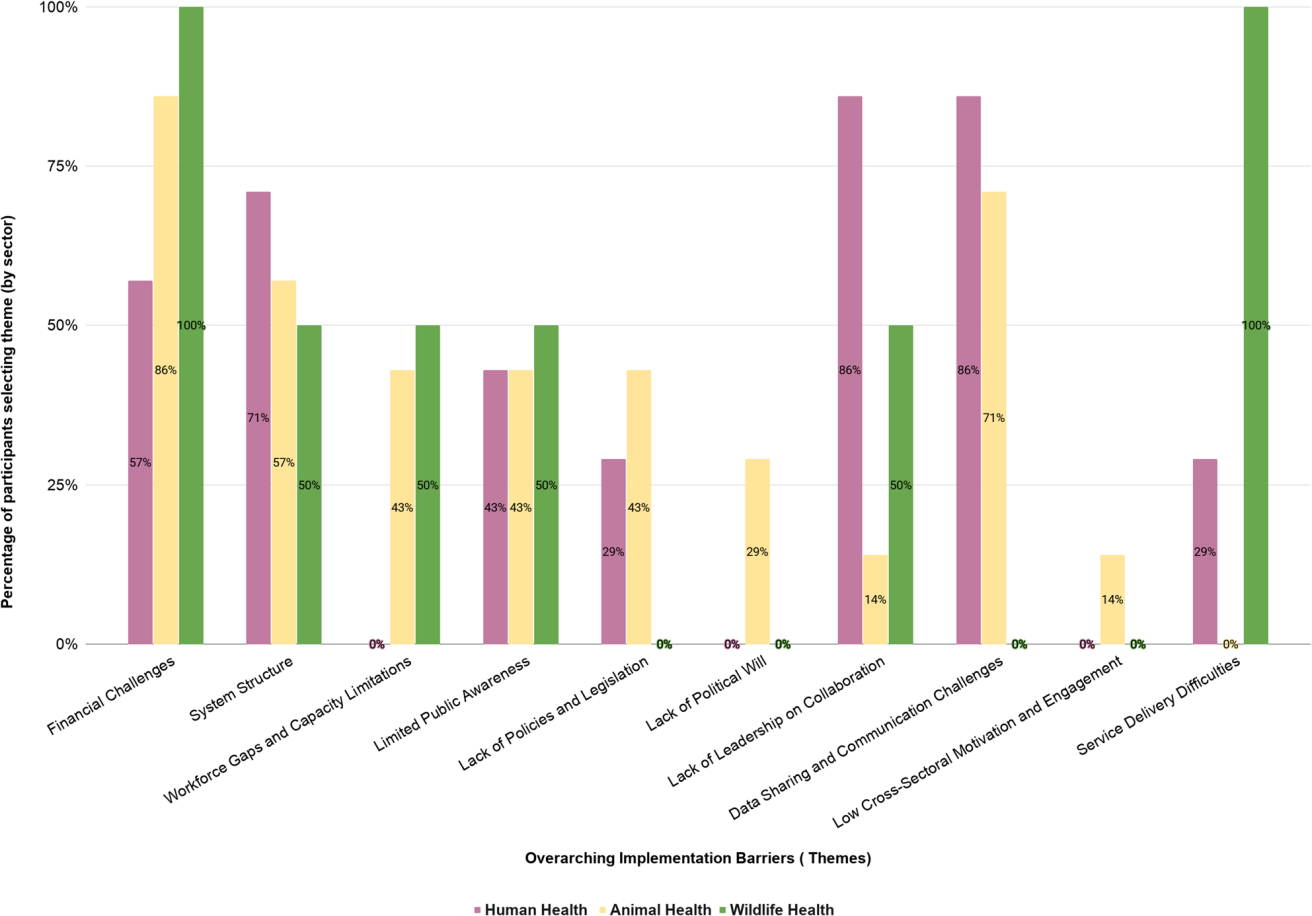
Sector-specific prioritization of overarching thematic barriers. Legend: This figure illustrates the percentage of participants in each sector - human health (*n* = 7), animal health (*n* = 7), and wildlife (*n* = 2), who selected each overarching thematic barrier as one of their top four priorities during the workshop

Overall, across sectors, participants’ voting reaffirmed the priority of Outer Setting and Outer/Inner Setting determinants. *Financial challenges* (12 votes, 75%), *data sharing and communication challenges* (11 votes, 69%), and *system structure* (10 votes, 63%) emerged as the highest-priority issues. Sectoral analysis showed both overlap and divergence. HH participants most frequently selected *lack of leadership on collaboration* and *data sharing and communication challenges* (86% each). AH participants prioritised *financial challenges* (86%) and *data sharing and communication challenges* (71%). WH participants unanimously selected *financial challenges* and *service delivery difficulties* (100%). Figure 1 presents the total number of votes received per theme across sectors and Fig. 2 shows the proportion of participants within each sector who selected each theme among their top four priorities.

### Group dialogue and evolving priorities

Participants engaged in a structured group discussion to reflect on sectoral differences and reassess priorities. The dynamic conversation, at times contentious, ultimately led to consensus on a shared top barrier. The discussion was structured around facilitator-posed hypothetical scenarios designed to probe deeper priorities beyond the initial voting results.

The discussion is presented in a sequence that reflects the evolution of priorities during the workshop. Within each thematic subsection, quotes are organised by the argument they illustrate rather than the precise order in which they were spoken. Selected quotes are lightly edited for clarity with full transcripts available in Additional File 3. All identifying information has been removed except first author references to preserve narrative authenticity and reflect the researcher’s embedded role in the study context.

### Diverging views on data sharing as a priority

Participants expressed surprise that *data sharing and communication challenges* ranked second. A WH actor challenges this, referencing successful national-level coordination:

My surprise is data sharing [.]During the Marburg outbreak two years ago, there was good data sharing between the environment, AH, and HH […] It may not be perfect in the 16 regions, but in some, these systems are working. We are not starting from zero (Q1, WH)

An AH actor acknowledged recent improvements but suggested they may not yet be widely visible (Q2, AH). Others, however, noted that sharing remains episodic and not embedded in routine workflows:

It works during outbreaks, but routinely it’s a problem. HH and AH sectors have separate reporting channels. Sometimes there’s information relevant to both groups, but because we are all following our channels, we are not able to know, so that we can respond. (Q3, HH)

### System structure emerges as the core implementation barrier

To probe deeper priorities, facilitators posed a hypothetical scenario: *“If money were no longer a problem, which of the remaining barriers would be most critical?”* Across sectors, participants overwhelmingly identified a weak *system structure* as the core implementation barrier, citing unclear mandates, a lack of coordination mechanisms, and fragmented institutional roles.

No. I’ll go for system structure […] if there was a proper structure, that data sharing and communication challenges wouldn’t exist. You can clearly see who the leader is; we will see our lines of collaboration. (Q4, HH)

AH participants described exclusion from key coordination platforms:

Even the district health management team, I’m not part of it […] during health meetings, I’m not called. So how do I report on AH activities? […] When I came to the district, I wanted data on dog bite cases. It took me 3–6 months before they gave it me. (Q5, AH)

Participants discussed how structural hierarchies limit AH visibility. Although VSD operates under the Ministry of Food and Agriculture nationally, at district level, they are nested within the Department of Agriculture, whereas GHS operates as parallel directorates. This structure impedes AH access to intersectoral platforms:

Because of decentralization, veterinary units at the municipal level is hanging. […] most assemblies only formally recognise Agric, not vet, unless they choose to invite us. It’s not that we don’t want to participate; it’s that the structure doesn’t include us. […] if we have the structure in place, … The person knows that automatically, the Agric director is not supposed to come, it’s supposed to be the vet (Q6, AH)

These hierarchies not only institutionally marginalized AH actors but also led to limited interpersonal contact and reliance on informal networks. One noted that they only actively sought out the new district vet after the research team’s visit during Phase 1:

My relationship with veterinary tends to be personal. When I first moved here, I never saw the vet. The first time was during a rabies death, I had to go find him […]. He helped, but when he went on retirement, I didn’t hear from him again. When the new person came, I didn’t see the person. It was only when Joannishka came when we went to find the new person. The person is divorced from the activities of the assembly. So if I don’t physically see the person, I wouldn’t even know them. Meanwhile, the Agric person is everywhere, so when it comes to zoonoses or OH, you think Agric, not vet. It’s not intentional, it’s structural.

(Q7, HH)

Additional insights on reporting misalignments, leadership gaps, and inactive committees were shared (Q2, Q8, Q9).

### Reintroducing financial challenges: still relevant, but not root cause

In the second scenario, participants were asked whether *financial challenges* should remain the top priority if current fiscal constraints remain unchanged. Participants still reaffirmed that structural and governance issues, not funding, were the root issue.

No. If systems are in place, we can find a way of getting the finances. If there is good collaboration and good leadership, wherever the money is, it will come (Q10, HH)

Currently, there’s something that is catching fire, ‘systems approach.’ […] Policy … , Leadership, funding and everything comes under it […] if you have your systems and structure right, everything goes on well. […] We may not need a particular funding basket for OH activities, [each sector] will still use the normal funding that they get, […] Systems is definitely number one (Q11, HH)

Others stressed the interdependence of finance and structure, and the role of political will:

If we don’t have the financial support, we cannot build a good system. […] They are interconnected and are managed by political will. […] We can have everything, but once the political head is not interested, it won’t work.

(Q12, HH)

Some emphasised that OH needs better structural alignment rather than new funding:

I have a contrary view. Currently, we are doing it. […] We don’t need large baskets of money. We just have to agree that, for instance, every month, [we will exchange reports] and it’s a structure. […] Money helps, but OH is not a strange thing. We [are] already doing it in different ways; we just have to put it all together. I still stand by structure … (Q13, HH)

AH participants highlighted resource inequities, warning that without addressing these, OH implementation would remain symbolic:

I don’t even have a computer […] how do I keep doing reports? For OH to work, each party has to be equally equipped. […] We can say we’ll share reports, but OH will not work unless we address the underlying issues. Financial [challenges] is a symptom, but the real cause is system structure, [which] is also linked with political will (Q14, AH)

Additional reflections on implementation bottlenecks, especially at the district level despite available funding, were shared (Q15).

### Policy and leadership embedded in system structure

Participants linked structural limitations to *absent or weak policy frameworks*, emphasizing the need for clearly codified mandates and formalised collaboration structures to ensure OH implementation is grounded in institutional clarity.

System structure is also determined by policy and legislation … it guides finances, system structure, leadership, and collaboration. […] without that, all these things will probably not even work. (Q16, HH)

Others noted that legal frameworks could synchronize siloed systems (Q17, WH) and that structures alone are insufficient without supporting policy to drive implementation (Q18, AH). Others also emphasised that how policies are developed, particularly stakeholder inclusion, is just as critical as their existence (Q19, AH).

### Re-ranking and emerging consensus

**Fig. 3:**
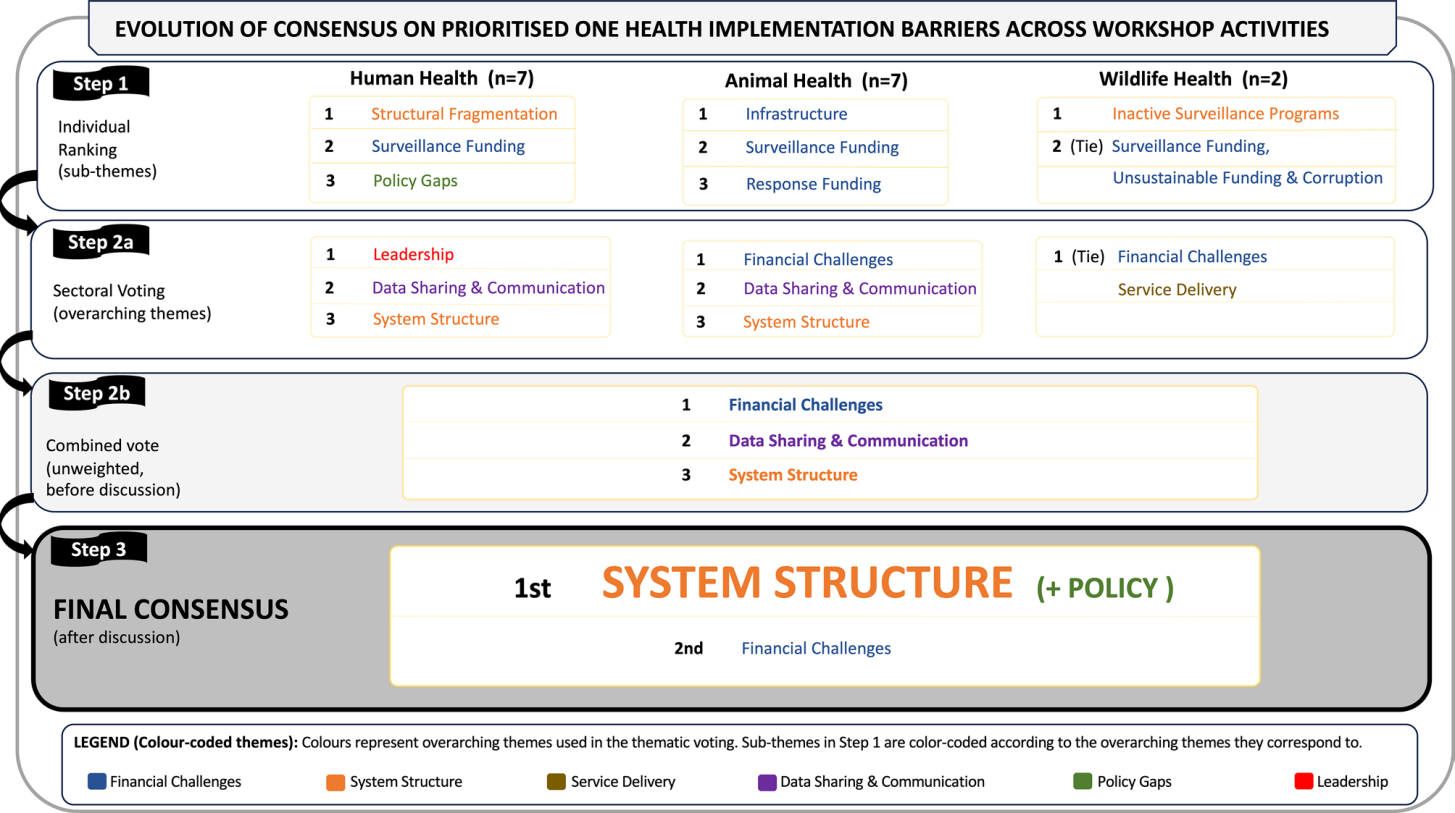
Evolution of consensus on prioritised One Health implementation barriers across workshop activities. Caption: The figure illustrates the multi-step consensus-building process used during the Phase 2 workshop. Step 1 presents the top-ranked sub-themes from the individual ranking exercise (Activity 1), colour-coded by their corresponding overarching themes. Step 2a (Activity 2) shows the top overarching themes selected through sectoral voting, while step 2b presents the combined (unweighted) vote across all participants prior to group discussion. Step 3 (Activity 3) shows the final consensus reached through facilitated intersectoral dialogue. Theme labels are abbreviated for clarity; full theme names are provided in Table 1. Sectoral sample sizes (**n**) are indicated in column headers to ensure transparency in representation

During the dialogue, participants requested a re-vote. Of 16 participants, 15 ranked *system structure,* combined with *lack of policies,* as the most important implementation barrier. *Financial challenges* ranked second, while *data sharing* was excluded. The evolution of consensus through structured individual ranking, thematic voting, and group deliberation is illustrated in Fig. 3.

### Recommendations from group work

In response to the prioritised barriers of *system structure* and *financial challenges*, participants were split into two groups to propose practical recommendations. While both groups were instructed to develop recommendations for the two prioritised barriers, time constraints resulted in Group 2 developing recommendations for the top priority barrier only, while Group 1 addressed both.

Both emphasised the need for clearer coordination frameworks across sectors. Group 1 proposed formal reporting guidelines, a communication policy framework and greater structural independence for VSD from the agricultural directorate. Group 2 proposed an interim Memorandum of Understanding (MOU) to guide information sharing while awaiting formal OH policies. Participants also highlighted workforce shortages (G1) and leadership training needs (G1), plus integration of OH actors into municipal health committees (G2).

While developed under “System Structure” and “Financial Challenges”, many recommendations reflect interconnected themes such as leadership, workforce capacity and policy gaps, highlighting OH implementation complexity. Full recommendations and their cross-cutting relevance are summarized in the analytical matrix below (Table 4).

**Table 4:** Participant group recommendations mapped to prioritised One Health implementation barriers (Workshop Activity 4)

Group	Primary Theme	Recommendation	Mapped Analytical Themes
Group 1	System Structure & Policies	Develop clear guidelines for reporting and communication between One Health sectors, supported by a formal policy framework.	Lack of Policies and Legislation, System Structure, Data Sharing and Communication Challenges
		Advocate for the independence of Veterinary Services from the Ministry of Agriculture to improve representation and reduce ambiguity in multisectoral committees.	System Structure, Lack of Leadership on Collaboration
		Establish clear human resource guidelines, especially for districts lacking technical staff; empower community-level health workers to support implementation.	Workforce Gaps and Capacity Limitations Service Delivery Difficulties
		Strengthen leadership through targeted capacity-building across sectors.	Lack of Leadership on Collaboration Workforce Gaps and Capacity Limitations
		Provide basic infrastructure and logistics, including offices and equipment, to support implementation.	Service Delivery Difficulties Financial Challenges
	Financial Challenges	Promote efficient use of existing resources (e.g., IGF for surveillance activities).	Financial Challenges
		Advocate for dedicated funding streams for One Health activities, equitably shared across sectors.	Financial Challenges Lack of Policies and Legislation
		Foster partnerships with international stakeholders for district-level logistical and financial support.	Financial Challenges Lack of Leadership on Collaboration
Group 2	System Structure & Policies	Develop an interim Memorandum of Understanding (MOU) among One Health sectors to formalize information-sharing while the national policy is still in development.	Lack of Leadership on Collaboration Lack of Policies and Legislation, Data Sharing and Communication Challenges
		Facilitate local stakeholder meetings to adapt and operationalize the MOU at the district/municipal level.	System Structure Low cross–sectoral motivation and engagement
		Integrate One Health stakeholders into Municipal Health Committees to ensure visibility, accountability, and alignment with district planning.	Lack of Political Will Low cross–sectoral motivation and engagement Lack of Leadership on Collaboration
		Accelerate the development and implementation of national One Health policy and/or legislation.	Lack of Policies and Legislation,
	Financial Challenges	Explore the establishment of a dedicated One Health fund for logistical support, while acknowledging feasibility constraints.	Financial Challenges

Legend: This table presents recommendations co-developed by participants during Workshop Activity 4. Participants were divided into two groups and generated recommendations for the two prioritized overarching implementation barriers: System Structure and Policies, and Financial Challenges. Many recommendations address multiple interconnected themes and are mapped to the corresponding cross-cutting analytical themes identified in the study

### Tensions between policy and practice

Participants debated whether integrating OH actors into health committees was legally permissible. One HH participant argued that this would require a legal amendment, as committee membership is policy-defined (Q20, HH). Others disagreed, noting that districts can co-opt members with leadership support:

No, in my district, we have co-opted some people to join the committee. We have the law, but if you think people are necessary […] you can co-opt. [However], your Municipal Chief Director must be interested and know why, because it also comes with a financial cost. (Q21, HH)

### Workforce shortages emerge as a cross-sector concern

Workforce capacity, particularly within the AH and WH sectors, was flagged as a critical implementation barrier largely underemphasised in group recommendations. A HH participant noted that some veterinary officers cover multiple districts.

Did any of the groups look at ‘workforce’? One veterinary officer is for [District A] and [District B]. It’s a challenge, you are looking for Dr. [name] and he’s at. [District B] (Q22, HH)

A WH actor then expressed disappointment, highlighting extreme workforce shortages in their sector:

I was very disappointed at the workforce gaps. In the whole country, we are just three. You can see my face, I already look old […] Jumping from the north to south, east to west … there’s no rest. (Q23, WH)

This exchange reaffirmed that workforce shortages are a critical implementation barrier across sectors, requiring greater emphasis on future recommendations.

## Discussion

### Key findings

As introduced in the Introduction, CFIR distinguishes between Outer Setting factors, such as governance structures, policy environments, and inter-organisational relationships, and Inner Setting factors related to organisational processes, leadership, and resource availability. CFIR conceptualises these domains as determinants of implementation, providing a lens for understanding how multilevel contextual factors shape OH operationalisation in Ghana.

Our analysis reveals that implementation barriers were predominantly external, with eight of ten themes (80%) classified as pure Outer Setting barriers, while two (20%) reflected interactions between Outer and Inner Setting factors. Crucially, no barriers were purely Inner Setting. Within the CFIR framework, this pattern indicates that implementation feasibility is primarily shaped by system-level Outer Setting determinants in the Ghanaian context.

A central finding of this study is how intersectoral dialogue reshaped stakeholder perceptions of core OH implementation barriers. Initially, HH, AH, and WH actors prioritised different barriers reflecting their distinct operational contexts. HH actors emphasised governance and leadership, consistent with their positioning within more formalised institutional structures where intersectoral coordination mandates remain unclear despite existing accountability frameworks. AH participants overwhelmingly highlighted financial constraints, with 86% selecting financial challenges and all three of their top-ranked sub-themes falling exclusively within the financial barriers category, reflecting how chronic resource precarity shapes sectoral perceptions of constraints. WH participants emphasised structural deficiencies, financial limitations, and service delivery gaps, reflecting the limited presence and operational capacity of wildlife surveillance at the district level.

Notably, some barriers were not uniformly experienced across sectors. Data sharing and communication challenges were highly prioritised by HH and AH participants but not by WH actors, while workforce and capacity constraints were disproportionately emphasised by AH and WH participants. These patterns highlight that while some barriers are shared, their relative importance and underlying drivers differ across sectors. The data sharing divergence is particularly instructive when examined through the lens of administrative level rather than sector alone. WH actors, primarily positioned at national and regional levels, did not prioritise data sharing as a key barrier during voting. The voting data revealed this divergence, while the subsequent intersectoral dialogue provided its explanation: national-level WH participants drew on their experience of functional data exchange during outbreak response, whereas district-level HH and AH participants described routine data sharing and communication failures that were less visible at higher administrative levels. This highlights how implementation barriers may be differentially experienced across system levels.

However, deliberation helped participants re-evaluate their views, reframing financial and data-sharing issues as symptoms of a deeper problem: the absence of a unified OH system. This reframing illustrates how Outer Setting constraints can shape, and in some cases mask, Inner Setting challenges, with structural governance gaps manifesting as operational barriers at the organisational level. This shift in perspective, captured in the consensus that “money will never be enough without structure,” underscores the primacy of governance over resource inputs. Ultimately, participants concluded that effective OH implementation requires coordinated policies, reporting systems, funding mechanisms, and accountability structures, rather than technical training or individual-level capacity building alone.

### “Weak system structure” – the central governance bottleneck

Participants identified the absence of a formalised OH system and supporting legal mandates as the central bottleneck for ZDSR in Ghana. Although progress has been made, such as the establishment of a national OHTWG and a planned OH policy, routine coordination remains limited. Intersectoral engagement is often reactive, triggered primarily during emergencies, with participants noting that collaboration ‘works during outbreaks, but routinely it’s a problem,’ a pattern consistent with findings from other contexts [31, 51, 52]. At district level, these structural barriers were more pronounced. AH and WH officers reported exclusion from Inner Setting decision-making platforms, delays accessing HH data, and lacking formal recognition within local governance structures. Separate reporting channels, incompatible mandates and uncoordinated systems further undermine routine collaboration.

These structural deficits represent governance failures rather than operational inefficiencies, with absent legal mandates, fragmented tools, and unclear leadership undermining accountability and institutional buy-in. For example, HH and AH data are not routinely shared under a common platform due to the lack of mandated protocols. This governance gap directly hinders timely response during outbreaks. These insights align with broader literature identifying governance fragmentation as a persistent OH implementation barrier across diverse health system contexts [5, 8, 12, 13], consistent with global frameworks highlighting the interdependence of leadership, governance, funding, and coordination [53]. Investing in OH surveillance infrastructure without clarifying leadership roles or data-sharing protocols would likely yield limited implementation success.

### Workforce architecture and the limits of coordination-based models

Beyond governance structures, workforce architecture emerged as a critical Outer Setting barrier. Extreme workforce imbalances (three wildlife officers nationally, individual animal health officers covering multiple districts), combined with structural exclusion from health coordination platforms, represent workforce planning failures that compromise disease surveillance capacity. Ghana’s OH implementation faces a fundamental paradox: policy frameworks assume coordination among roughly equivalent sectoral actors, yet these imbalances create operational infeasibility, rendering conventional peer-coordination models structurally impossible.

From a health services perspective, coordination carries transaction costs: meetings, communication, joint planning, and information exchange all require time and personnel [40, 54]. When workforce ratios are severely imbalanced, these transaction costs become prohibitive. Individual officers covering multiple districts cannot sustain routine engagement across numerous facilities and personnel. Participants adapted through personal relationships rather than institutional protocols, reducing transaction costs through selective engagement. However, such adaptations create fragility: collaboration dissolves when personnel change, and sectors with greater workforce presence (e.g., agriculture) become default partners despite lower technical relevance for ZDSR.

This workforce imbalance is entrenched by separate ministerial control. HH operates under the Ministry of Health, AH under the Ministry of Food and Agriculture (nested within agricultural departments at district level), and WH under the Forestry Commission. Workforce planning for multisectoral health services thus occurs in fragmented bureaucratic silos, with no mechanism for coordinated deployment based on OH service delivery needs. Even with perfect coordination structures at district level, workforce ratios remain dictated by separate ministerial priorities. Strategic workforce investment for OH implementation, therefore, requires cross-ministerial governance mechanisms [5].

Effective health workforce planning for zoonotic disease control must extend beyond traditional human health cadres [8, 39]. These findings suggest that OH implementation in contexts with extreme workforce imbalances requires coordination models that differ from peer-coordination frameworks developed in more balanced systems, an area that remains underdeveloped in the current One Health and implementation science literature. Although *workforce* was not included in participants’ formal recommendations addressing *system structure* and *financial barriers*, it was subsequently raised as a critical omission, suggesting workforce constraints may be viewed as intractable realities beyond immediate policy interventions. Yet without addressing them, even well-designed governance frameworks face operational impossibility.

This workforce-coordination mismatch extends beyond OH to other multisectoral health initiatives in resource-constrained settings [40]. Health services research must develop coordination models designed for workforce imbalances, not workforce parity. The assumption underlying many multisectoral health frameworks that creating coordination structures will enable collaboration [53, 55] fails when workforce architecture creates transaction costs that exceed feasible capacity.

### Implications for policy, strategy and capacity building

This study offers important insights for OH policy and implementation in Ghana. Building on participant-generated recommendations (Table 4), several actionable strategies can support early-stage implementation within existing institutional structures.

In the short term, stakeholders emphasized the development of interim Memoranda of Understanding among OH sectors to formalize information sharing and coordination while the national OH policy is still under development, alongside the establishment of clear reporting and communication guidelines. Participants also recommended integrating animal and wildlife health actors into Municipal Health Committees and facilitating regular local stakeholder meetings to operationalize coordination at the district level, ensuring that national-level frameworks are translated into practical operational arrangements.

A structurally significant recommendation from participants was the advocacy for greater clarity in the representation of the Veterinary Services Department in multisectoral spaces, independent of its administrative placement under the Ministry of Agriculture. While full administrative separation may present structural and political challenges in the short term, the underlying problem, that decentralization has rendered veterinary units invisible in intersectoral spaces where assemblies formally recognize agriculture rather than veterinary services. This creates a misalignment between administrative representation and technical expertise in multisectoral decision-making and can be addressed through a more targeted intervention. The planned OH policy presents an opportunity to explicitly distinguish veterinary technical roles from broader agricultural administrative representation in committee membership guidelines, ensuring that OH-relevant intersectoral spaces mandate veterinary officer participation rather than defaulting to agricultural directorate representation. Beyond this, a systematic audit of existing intersectoral committee membership guidelines is recommended to identify where specifications currently default to district agriculture directors and to determine whether district veterinary officers are the more appropriate representatives for zoonotic disease and OH matters.

In parallel, participants identified the need for targeted workforce and capacity interventions, including the development of human resource guidelines for underserved districts, strengthening leadership through cross-sectoral capacity building, and leveraging community-level personnel to support implementation in areas where sectoral officers are absent. Ghana’s current wildlife health workforce of three veterinarians nationally is insufficient for meaningful OH participation at the operational level, while AH actors are frequently spread thin across multiple districts, further limiting their capacity for routine intersectoral engagement. The planned OH policy should therefore incorporate explicit workforce investment targets for both animal and wildlife health sectors, with deployment strategies that reflect OH surveillance needs across geographic and administrative levels rather than single-sector priorities.

Financial recommendations focused on both optimizing existing resources and developing dedicated funding mechanisms, including the efficient use of Internally Generated Funds for surveillance activities, the establishment of OH-specific funding streams equitably shared across sectors, and fostering partnerships with international stakeholders for district-level logistical and financial support. While participants recommended dedicated funding streams, a single centralized OH fund may face significant feasibility constraints given current resource limitations across all sectors. A more immediately pragmatic approach may be ensuring each sector maintains adequate resources for its own surveillance activities while formal coordination structures reduce the transaction costs of joint work. This would enable sectors to combine existing resources for specific collaborative activities without requiring fully integrated budgets. Evidence from cross-sectoral co-financing models suggests that effective resource pooling is facilitated by clear alignment of sectoral objectives and roles, and is typically supported by formal governance and accountability structures that enable coordination across sectors [56]. Future participatory processes involving both district and national stakeholders should explicitly deliberate on financing mechanisms appropriate to Ghana’s governance and fiscal context.

Finally, while technical training remains essential, as demonstrated by initiatives such as the Field Epidemiology and Laboratory Training Programme [22, 57], the workforce architecture and governance challenges discussed above must be addressed concurrently. From a health services research perspective, these findings underscore that sustainable OH implementation requires integrated policy and organizational reforms spanning governance structures, workforce planning, and financing mechanisms [5, 8, 58], and that technical capacity building is most effective when implemented alongside, rather than in place of, these structural reforms. Ghana’s planned activities, finalizing OH-informed policies and boosting multisectoral cooperation, are well-aligned with these stakeholder-identified priorities [59, 60].

### Regional and global context

Ghana’s governance-related barriers reflect broader regional patterns observed across both regional and global contexts. In Nigeria, the national OH strategy introduced in 2019 continues to face poor coordination and sectoral tensions [61, 62]. Uganda’s more developed platform still struggles with inconsistent commitment and limited enforcement [63]. Similarly, reports from the Economic Community of West African States highlight legal ambiguity, fragmented financing and weak cross-sector accountability [64].

Importantly, these challenges are not confined to resource-limited settings. Evaluations of OH surveillance systems across multiple European countries found that professional silos, lack of operational and shared leadership, limited data sharing, and absence of harmonised indicators contributed to suboptimal OH surveillance functioning [12]. Broader analyses of global One Health governance similarly identify sectoral, professional, and institutional silos, fragmented legal and regulatory arrangements, and crisis-driven financing as persistent barriers to effective implementation across diverse country contexts [13]. Together, this evidence suggests that Ghana’s barriers are consistent with structural governance challenges that can persist across income levels, rather than being driven solely by resource limitations.

Consistent with this, evidence from both high- and low-income settings highlights common enabling conditions for effective One Health collaboration. A scoping review identified formal intersectoral structures with explicit mandates, clear leadership frameworks, and inclusive stakeholder engagement processes as key enablers of sustained collaboration across settings [65]. Additional evidence suggests that One Health efforts are less effective when perceived as led by a single ministry rather than shared across sectors [5], underscoring the importance of the tripartite structure Ghana has established through its OHTWG as a foundation for shared ownership and sustained collaboration.

These insights suggest that Ghana is well positioned to formalise coordination mechanisms and clarify cross-sectoral mandates at its current policy formalisation stage, drawing on documented international experience.

### Contributions to the literature

Most OH implementation research focuses on conceptual frameworks or disease-specific programmes [33, 35], with limited empirical attention to operational-level governance barriers faced by frontline implementers [5, 8]. This study addresses this gap, responding directly to calls for more research on OH governance [66], by examining how operational-level actors across HH, AH, and WH sectors in Ghana prioritise operational constraints through a participatory, barrier-focused CFIR-guided analytical process.

The application of CFIR as a determinant framework enabled differentiation between system-level (Outer Setting) and organisational-level (Inner Setting) influences on implementation, revealing that 80% of high-priority barriers were situated in the Outer Setting and perceived as underlying drivers of Inner Setting challenges. This finding challenges approaches that prioritise organisational capacity strengthening or technical training as the main route to enabling OH operationalisation [8, 33, 35, 55]. Our results indicate that systemic Outer Setting barriers constitute the principal obstacles in the Ghanaian context, overshadowing organisational resource constraints.

Methodologically, this study demonstrates the value of structured intersectoral dialogue for surfacing systemic constraints. Unlike sector-specific assessments that often overlook cross-cutting issues, our participatory approach revealed how stakeholder priorities shift from initial resource concerns to a consensus on governance gaps when sectors deliberate collectively. This methodological finding, that collective dialogue can surface systemic barriers and foster cross-sectoral consensus, has important implications for health system reform processes requiring multisectoral buy-in [38]. Furthermore, this study highlights how administrative level, not just sector, shapes stakeholder perceptions of implementation barriers. WH participants operating at the national and regional level did not identify data sharing and communication as a priority concern, while district-level HH and AH participants ranked it among the highest barriers. This divergence suggests that OH policy consultations relying exclusively on national-level representation risk underrepresenting operational barriers experienced at the district level, a finding with direct implications for how stakeholder engagement processes are designed in future OH implementation research and policy development.

Finally, beyond OH, these findings inform health services research on implementing complex, multisectoral initiatives. The governance barriers, coordination failures, and workforce planning gaps identified here are directly relevant to other delivery models, such as planetary health [67] and integrated care [68, 69], that rely on sustained intersectoral collaboration.

### Limitations and future research

This study has some limitations. As a qualitative exercise, our findings highlight context-specific barriers within three regions and may not be fully transferable to all contexts across Ghana. Other stakeholders (e.g., the private sector or community representatives) representing other aspects of the Outer Setting may have offered different insights. While the participatory workshop supported open dialogue, certain individuals or sectors may have shaped the discussion more than others.

Although all WH officers were interviewed and they oversee surveillance nationwide, the absence of WH personnel at the district level limits the granularity of implementation insights from that sector, particularly on how the Outer Setting policy context translates to the Inner Setting at the local level. Additionally, the unequal sectoral representation in the prioritization workshop (7 HH, 7 AH, 2 WH participants) means that combined weighted scores were predominantly shaped by HH and AH perspectives. This reflects the actual workforce distribution within Ghana’s ZDSR system rather than a sampling bias, as the two WH participants represented complete national-level WH coverage available for the study.

A key limitation of this study is that the identified barriers were predominantly located within the Outer Setting domain of CFIR. This likely reflects Ghana’s current stage of OH development, where formal policy frameworks are still being established and implementation has not yet been institutionalised at the organisational level. As a result, Inner-setting factors, such as organisational culture, leadership practices, and workflow integration, may not yet be fully observable.

Future research could build on these findings by applying implementation frameworks that explicitly account for policy temporality and pre-implementation dynamics, such as the EPIS framework [70] or the policy-focused implementation framework proposed by Bullock et al. [71]. Additionally, adaptations of CFIR for LMIC contexts, such as that proposed by Means et al. [72], may offer further analytical depth in examining how structural and contextual factors shape implementation processes in similar settings.

This study did not quantify the frequency of implementation barriers, and therefore, findings highlight shared priorities, not national prevalence. Future research could use surveys or real-time case studies of zoonotic outbreaks to test which structural and operational gaps most directly affect response outcomes. Additionally, longitudinal studies tracking OH implementation progress and comparative studies across different African contexts could provide valuable insights.

As CFIR was applied analytically following data collection rather than used to inform the interview guide prospectively, three of its five domains were not explicitly explored with participants. Characteristics of Individuals was outside the study’s focus on systemic and structural barriers; Implementation Process was not applicable given that formal OH implementation had not yet commenced; and Intervention Characteristics, including perceptions of OH’s adaptability, complexity, and cost, were not elicited despite being relevant to a pre-implementation context. Future studies applying CFIR prospectively from the design stage would be better positioned to examine these constructs alongside the system-level determinants reported here.

Despite these limitations, the study offers a grounded picture of the implementation barriers Ghana faces in operationalising OH. The findings present actionable priorities, developed through cross-sector dialogue, that can support stronger coordination, clearer legal frameworks, and more resilient surveillance systems.

## Conclusions

Most barriers to operationalising OH in Ghana’s ZDSR system were system-level, reflecting gaps in governance, coordination, and enabling policy frameworks. Through participatory dialogue, stakeholders reframed financial and data sharing challenges as symptoms of deeper structural problems, collectively prioritizing solutions focused on governance reform, mandate clarification, and data system interoperability. While technical investments remain important, sustainable progress requires systemic reforms that align legal, institutional, and operational components across ministries.

Beyond OH, these findings illuminate health system governance challenges facing multisectoral health initiatives across settings, particularly in LMICs where structural constraints may be more pronounced. The structural barriers, workforce architecture constraints, and coordination deficits identified here constrain effective service delivery across domains requiring intersectoral collaboration, from antimicrobial resistance to climate-sensitive diseases to pandemic preparedness. As health systems increasingly recognise that complex health challenges transcend sectoral boundaries, understanding these governance barriers becomes essential.

This study demonstrates that even well-designed coordination structures face operational impossibility when workforce architecture and policy frameworks remain fragmented across ministerial silos. The participatory approach employed here, which surfaced stakeholder consensus on root causes through structured dialogue, offers both methodological and empirical insights for health system reforms requiring sustained intersectoral coordination.

## Electronic supplementary material

Below is the link to the electronic supplementary material.

Supplementary material 1

Supplementary material 2

Supplementary material 3

## Data Availability

All anonymized data generated during this study that do not compromise participant confidentiality are included in this published article and its supplementary information files [Additional files 1, 2, and 3 containing supporting quotes]. The full interview transcripts are not publicly available due to participant confidentiality, but anonymized excerpts are available from the corresponding author on reasonable request.

## Abbreviations

AH: Animal Health
CFIR: Consolidated Framework for Implementation Research
COREQ: Consolidated Criteria for Reporting Qualitative Research
GAMA: Greater Accra Metropolitan Area
GHS: Ghana Health Service
HH: Human Health
KMA: Kumasi Metropolitan Area
LMICs: low- and middle-income countries
MESTI: Ministry of Environment, Science, Technology, and Innovation
MOU: Memorandum of Understanding
OH: One Health
OHTWG: Ghana One Health Technical Working Group
TAMA: Tamale Metropolitan Area
VSD: Veterinary Services Department
WD: Wildlife Division of the Forestry Commission
WH: Wildlife Health
ZDSR: Zoonotic disease surveillance and response
