# Supplementary material for: Stakeholder identification and prioritization of barriers to One Health implementation in Ghana’s zoonotic disease surveillance and response system: a sequential mixed-methods study
Source: BMC Health Serv Res. 2026 May 29;26:770. doi: 10.1186/s12913-026-14819-1 (PMC13221755; doi:10.1186/s12913-026-14819-1)
Supplement: Supplementary file 1 — Supplementary material 1 [file 12913_2026_14819_MOESM1_ESM.pdf]

Additional file 2: Supporting Quotes for Overarching Themes (Phase 1).

To maintain participant confidentiality and anonymity, quotes are attributed only by sector—HH (Human Health), AH (Animal Health), and WH (Wildlife). Quotations have been lightly edited for clarity, flow, and to remove repetition, while preserving the original meaning and voice of the speaker.

Theme	Label	Selected supporting quotes
Theme 1	Financial Challenges	*"What I think will be difficult for us is to have more resources to collect data to share with the One Health Technical Working Group ...And why don't we have the data? Because of lack of resources to collect it." (WH)*<br><br>*"Money ...that is always the problem that makes it difficult for us to collaborate. Especially when there's small money attached to it, and one is aware. When we collaborate, that's small money we'll share. [and] that sharing becomes a problem. ...I end up doing it on my own, they also end up doing it on their own [to] keep it to themselves [...]. All will boil down back to money" – (HH)*<br><br>*"I think it's funding…When there is an outbreak and you want to move out, who provides the funding?... One person will have to take the lead and pull the others along. But if you are waiting for that common fund you may never know." (HH)*
Theme 2	System Structure Challenges	*"The surveillance system does not support collaboration" (HH)*<br><br>*"The issue is that we are running different structures ...The vet structure is completely different from our side... We have the community, chip zones, sub districts and districts. We have structures at this level. The vets have districts and zones...That's it, they do not have any smaller structure. Even, their zones are very big ... And then it becomes very difficult, [when] collaborating at the zonal level" (HH)*<br><br>*"GHS has the Disease Surveillance unit. Everything that goes on in GHS [and] entered into their system, Disease Surveillance [sees] ...it's not the same as VSD. GHS [uses] EPI Zones. We should also zone to mirror the GHS zones. [That way], we can be on the same platform using the same tools. VSD may not have staff in some of the zones, but it's better we design our zoning according to GHS'. If we want to achieve proper One Health then, model our reporting system like [theirs], because they are far advanced. We should*

		copy them and paste. It's the same Ghana, […]so when they are talking about disease outbreak in zone A, it's the same thing with Veterinary in zone A…then OH can work." (AH)
Theme 3	Data sharing and communication challenges	*"One issue is availability of the data generated by the various disciplines… For instance, here, we don't actually know what is going on at the veterinary; the number of cases, the dog bites. We don't know that, and they also don't know what we have here." (HH)*<br><br>*"Because some of the data or some of the information, nobody wants it to move from their sector to another […] But if we made everybody to be aware that we are one […] If the understanding is there, it can work everywhere." (HH)*<br><br>*"Our weakness will be - We do not have a common platform that we communicate on. […] Within the sector and also with the Vets. […] If there's a common platform, there's no need for me to contact him or her, he will know. […] So there should be a common platform where we can all share ideas and information." (HH)*
Theme 4	Lack of Policies and Legislations	*"The roles are not even spelt out …That is why a disease control officer can say he is going to follow up on a dog case […] if they see a rabid dog can they see it? [...] can they confirm a rabies case?" (AH)*<br><br>*"Environment, has always been a difficult sector because we don't know who is the Environment. They're not well defined … We don't know who is the focal point. [At] the local government, they are different, [at] the national, they are different." (AH)*<br><br>*"[The collaboration] should be backed by the law, because without the law, it is toothless." (WH)*<br><br>*"Another issue is that these are professionals from different backgrounds. So if the lines are not well drawn, then it could be a failure. The conflict will actually lead to failure." (HH)*

Theme 5	Lack of Leadership on Collaboration	*"Leadership … because once you're bringing two sectors together, somebody has to lead." (HH)*<br>*"There's nothing that forces any collaboration at all, even the things that are laid down in policy, there's nothing that forces you, can decide to do it or not…it's up to you. […] The implementation is another thing. There are so many things on policy, but the implementation is not being done as supposed to be done. Policy for me is just paper, it is the practicalor the operationalization of it…" (HH)*<br><br>*"We have not had a champion; a lead institution to champion One Health. If you just say one health, one health, and [that] Ghana Health Service should take the lead. Yes, they don't want to. You know why? Because all identified diseases are not their diseases. WHO is not going to fund those things…WHO is the richest of the three … they should lead, but they cannot lead the content technically." (AH)*
Theme 6	Lack of Political Will	*"They are not interested, even to vaccinate dogs, you need enforcement. People do not see the reason why local dogs [matter] … you need enforcement from the assembly to do some of these things. (HH)*<br><br>*"Funds not being approved. Since I came here, we've not had the opportunity to conduct vaccinations but every year, they take monies for that… the money goes to the assembly but it doesn't get to us… less attention is given to vet issues" (AH)*<br><br>*"Commitment from the assembly …that's one of our weaknesses." (HH)*<br><br>*"In Ghana, something [new] comes in and then we're all [excited]. If we like, we'll do it but in no time, that regime is gone or another government comes in and all those documents are kept aside. The governmental changes do not allow it to function." (AH)*
Theme 7	Limited Public Awareness	*"Community members play a key role… If they do not report, we won't know. There was a case where a dog bit a man, and it ended up at the police station because the owner refused to provide vaccination records. People don't even know where to report. Some think things like cat bites aren't serious. But these are the people who know which dogs belong to who, where they sleep … they know the details, and they need to be part of this system." (HH)*<br><br>*"It's societal, it's cultural…We don't care about animals. You'll see a child with a stick hitting his pet in their house. They don't care..."(HH)*

Theme 8	Workforce Gaps and Capacity Limitations	*"I can speak for veterinary because they're always complaining, they don't have money, they don't have support, and they don't have the staff to do the work." (HH)*<br><br>*"We have untrained human resource for this type of activities. Apart from us vets, who are not everywhere, we can use the rangers and law enforcement activities to collect surveillance data, but they are not trained for that. We need stronger collaboration between our protected areas and the district vet offices"(WH)*
Theme 9	Service Delivery Difficulties	*"When they come for the vaccine it is not for free… at the rural level. And that is where the problem comes from, because they don't have the money. You may have people who have about 10-15 dogs, they use them for hunting, ... but they can't even afford a dose of GHC 3." (AH)*<br><br>*"Now the vaccines are sold everywhere…When you go, they won't even send you to a vet. They'll just sell the vaccines to you …They have given the opportunities to anybody who operates a pharmacy to get that vaccine and sell it at his or her own cost…It's bad to refer a patient to a pharmacy… 'go get a vaccine bring it to me, let me inject'. It's not done anywhere but this is what the health facilities are doing." (AH)*
Theme 10	Low Cross-Sectoral Motivation and Engagement	*"We also have a public health emergency team that has been dormant for a very long time that I need to reactivate. But then again, it comes to resources and commitment from us, I must be honest" (HH)*<br>*"Because they don't see the importance. They don't see the need. But if everybody comes to see the need … it would be for the benefit of all." (HH)*<br>*"In Accra here, it is very, very difficult to get dedicated community surveillance volunteers. Everybody is working for money, so… the volunteerism is becoming a challenge. It is a challenge." (HH)*<br>*"There's some form of laissez-faire attitude, not necessarily from our side, but ..., I've had some encounters especially with Rabies, they go there, instead of them to just follow the normal protocol, 'has the dog been vaccinated?' They don't necessarily do that. They just kind of leave the case and then the patient has to go and find a way he or she will get [the vaccine]" (AH)*
