# Supplementary material for: Stakeholder identification and prioritization of barriers to One Health implementation in Ghana’s zoonotic disease surveillance and response system: a sequential mixed-methods study
Source: BMC Health Serv Res. 2026 May 29;26:770. doi: 10.1186/s12913-026-14819-1 (PMC13221755; doi:10.1186/s12913-026-14819-1)
Supplement: Supplementary file 2 — Supplementary material 2 [file 12913_2026_14819_MOESM2_ESM.docx]

**Additional File 3: List of 35 Specific Implementation Barriers (sub-themes) and Raw Ranking Results from Phase 2 Activity 1**

This table presents 35 specific challenges identified from Phase 1 interviews. In Phase 2 (Activity 1), participants were asked to rank their top 10 most pressing challenges from this list with Rank 1 indicating the most important and Rank 10 the least important.

|  | **Specific Challenge** | **Explanatory Examples** | **HH 1** | **HH 2** | **HH 3** | **HH 4** | **HH 5** | **HH 6** | **HH 7** | **AH 1** | **AH 2** | **AH 3** | **AH 4** | **AH 5** | **AH 6** | **AH 7** | **WH 1** | **WH 2** |
| --- | --- | --- | --- | --- | --- | --- | --- | --- | --- | --- | --- | --- | --- | --- | --- | --- | --- | --- |
| **1** | Lack of Infrastructure | Lack of appropriate facilities (physical office, clinics, inadequare office space etc) |  |  |  |  |  | 2 |  | 1 | 1 | 7 | 2 | 1 |  | 2 | 4 |  |
| **2** | Unsustainable funding sources | Funded projects by external donors collapse as soon as funding runs out |  |  | 6 | 1 | 8 | 3 |  |  | 7 | 1 |  |  |  | 3 | 7 | 5 |
| **3** | Lack of funds/logistics for surveillance activities | Inadequate funding for surveillance activities. release and timing issues |  | 3 |  | 2 | 6 | 1 | 2 | 5 | 4 | 2 | 9 | 2 | 1 | 4 |  | 1 |
| **4** | Lack of funds/logistics for response/control activities | No funds to conduct vaccinations |  |  | 7 |  |  |  | 3 | 4 | 5 | 5 |  | 3 | 2 | 5 |  |  |
| **5** | Difficulty Generating Revenue | Inability to take money from community |  |  |  |  |  |  |  |  |  |  |  |  |  |  |  |  |
| **6** | Corruption | Money released for activites/vaccinations but does not get to actor. |  |  |  | 3 |  |  |  |  | 10 |  |  |  |  | 7 | 1 |  |
| **7** | Health System Structure not including collaboration | Differing system structures that do not mandate collaboration |  | 2 | 3 |  | 2 | 4 | 1 |  |  |  | 3 | 4 |  |  | 2 |  |
| **8** | OH actors missing from Public (Health) Committees | Vet not a member of health committees in some districts |  |  |  |  |  |  |  | 3 |  |  |  |  |  |  |  |  |
| **9** | Inactive Surveillance programs | Wildlife surveillance not too well organised |  |  |  |  |  |  |  |  |  | 4 |  | 6 |  |  | 3 | 2 |
| **10** | Relationship Issues | Vet under the Agriculture department not helping |  |  |  |  |  |  |  | 2 | 3 |  |  |  | 7 |  |  |  |
| **11** | Bureuacracy | Difficulty getting to an actor or data in another sector |  |  |  |  |  |  |  |  | 8 |  |  |  |  |  |  | 6 |
| **12** | Inadequate and/or lack of staff | Not enough staff which limits capacity. Some districts dont have vet staff |  | 9 |  | 4 |  |  |  |  |  | 3 |  |  | 3 | 6 |  | 4 |
| **13** | High turnover of actors | High turnover of actors causes disruptions in informal collaborative efforts |  |  |  |  | 10 |  |  |  |  |  |  |  |  |  | 5 |  |
| **14** | Low visibility and impact of actors | -Presence of vets not known by other sectors and community. not aware of relevance. -Not knowing new focal persons when an older actor retires |  |  | 8 |  |  | 5 | 4 |  |  | 6 |  |  | 4 |  |  | 10 |
| **15** | Unmotivated Community Volunteer staff | Difficulty getting dedicated community surveilance volunteers |  |  |  |  |  |  |  |  |  | 8 |  |  |  |  | 6 |  |
| **16** | Weak technical capacity of actors | -No regular inservice training-- to build capacity.  -National level actors having to step in to control outbreaks |  | 4 |  | 5 |  | 6 |  |  |  | 9 |  |  | 5 |  |  | 3 |
| **17** | Lack of Knowledge (actors)(1.knowledge in zoonosis.2. Public Health knowledge 3.Understanding of OH | -Inadequate knowledge on zoonotic diseases - Actors with no public health training, tend to not see the need to collaborate with other public health institutions -All actors/ sectors not understanding need to collaborate, understanding the One Health concept |  |  |  |  | 1 | 7 |  |  |  | 10 | 1 | 5 |  | 1 |  |  |
| **18** | Lack of Knowledge (Community) | Community members do not always report relevant information or are not educated on the need to |  |  | 4 |  |  |  | 10 |  |  |  |  |  |  | 10 |  |  |
| **19** | Lack of OH Policies and Legislations - General | - Lack of policies to guide relationships | 1 | 1 |  |  | 3 |  | 9 | 6 |  |  |  | 7 | 9 |  |  | 8 |
| **20** | Unclear Sector Roles | - No clarity on/ undefined roles . encroachment on each others' domains. | 2 |  |  |  |  |  | 5 |  |  |  | 4 | 8 | 10 |  |  |  |
| **21** | Lack of Political will | -Little to no support from district assemblies. lack of committment from them | 3 | 10 |  | 10 |  |  |  | 7 | 2 |  | 5 |  | 6 |  |  |  |
| **22** | No OH Leadership | Who is the champion or lead institution for OH? | 4 |  | 1 |  | 4 |  |  | 8 |  |  |  |  |  |  |  |  |
| **23** | Poor implementation strategies | Lots of talk about One health but no action. | 5 | 7 | 2 |  | 5 |  |  |  |  |  |  |  |  | 9 |  | 9 |
| **24** | Poor data storage/management | No passing down of records. poor date storage practices |  |  |  |  |  |  |  |  | 9 |  |  |  |  | 8 |  |  |
| **25** | No uniformed data collection protocols | No uniform data collection protocols for the zoonotic diseases | 6 | 6 |  |  | 7 |  |  |  |  |  |  |  | 8 |  | 9 |  |
| **26** | No joint communication platforms | No uniform platform for communication |  |  |  | 9 |  | 8 | 6 |  |  |  | 10 | 9 |  |  |  |  |
| **27** | Differing reporting/data sharing platforms between sectors | Differing reporting and data sharing systems between sectors. one system more advanced than other | 7 | 5 | 5 | 8 | 9 |  |  | 9 |  |  |  |  |  |  |  |  |
| **28** | Difficulty with accessing & availability of other sector's data | Difficulty with access and availability of data generated by other sectors | 8 |  |  |  |  |  | 7 |  |  |  |  | 10 |  |  | 8 |  |
| **29** | Other sectors' not open to data sharing | Some sectors not always open/willing to share data | 9 |  |  |  |  |  |  |  |  |  | 6 |  |  |  |  |  |
| **30** | Poor data sharing practices | No routine sharing of data between ghs & vet |  |  |  |  |  | 9 |  | 10 |  |  |  |  |  |  |  |  |
| **31** | Lack of assertiveness of actors | Some actors not proactive in getting information from other sector |  | 7 |  |  |  |  |  |  |  |  |  |  |  |  |  |  |
| **32** | Inferiority complex | Some sector's feel they are undermined by others |  |  |  |  |  |  |  |  |  |  | 7 |  |  |  | 10 |  |
| **33** | Each system has a different objective | Surveillance systems of human, animal and wildlife not having common goals |  |  | 9 | 7 |  | 10 | 8 |  |  |  |  |  |  |  |  |  |
| **34** | Low interest in collaborating | - Low interest in each sectors activities.  -Some of the phemc are dormant due to lack of lack of committment | 10 |  |  |  |  |  |  |  | 6 |  | 8 |  |  |  |  |  |
| **35** | Difficulty getting needed drugs and vaccines | Challenges getting rabies Post Exposure Prophylaxis Vaccines |  |  | 10 | 6 |  |  |  |  |  |  |  |  |  |  |  | 7 |

NB: HH stands for human health, AH stands for animal health, WH stands for wildlife health)
