# Supplementary material for: Stakeholder identification and prioritization of barriers to One Health implementation in Ghana’s zoonotic disease surveillance and response system: a sequential mixed-methods study
Source: BMC Health Serv Res. 2026 May 29;26:770. doi: 10.1186/s12913-026-14819-1 (PMC13221755; doi:10.1186/s12913-026-14819-1)
Supplement: Supplementary file 3 — Supplementary material 3 [file 12913_2026_14819_MOESM3_ESM.pdf]

Additional File 4 – Participant Quotes from Workshop Activity 3 & 4: Intersectoral Dialogue and Recommendation Development

Quote ID	Theme/Sub Theme	Quote (Full Text)	Sector
Q1	Data Sharing Surprise	*“No, my surprise here is data sharing and communication challenges. I don't know where people are, but with the outbreak of Marburg virus diseases just about two years ago, I realised that there was good data sharing invitations especially between the environment, animal health and human, and that led to very good field investigations, collection of data and then all the communications that were made up… Yeah. So I'm just talking in general looking at what is happening on the One Health Technical group that over the years, just like what she was saying, we used to see maybe reporting of zoonosis diseases and those kind of things just like the example I was giving of the Marburg virus disease. But fast forward this year, I have seen that there is a platform that shares disease incidence information between the Veterinary Services and Ghana Health Services. So every week, the National Epidemiology unit from that veterinary services share incidences to the one health working group platform, I think that is a plus. Maybe it's not all of us that are on that platform that see what comes up every day. Now, my senior was drawing my attention to the fact that that is at the national but what I keep saying is that if we don't have a Vet at the district level, there will be nothing for the national level to report, do you understand? So those of you who have worked in the district, when there are incidents of rabies cases, I think this time round Ghana Health Service and Veterinary service at least try to collaborate and then send the reports on that. It may not be perfect in the 16 regions and then the 100 and something districts in Ghana, but at least in some regions, like Ashanti Region and other places these systems are working. So we are not just starting from zero, at least we have moved somewhere and I think that it’s a big plus. That's why I was surprised to see this.”*	Wildlife Health

Q2	Data Sharing Surprise & System Structure emerges as the core challenge	*"I was actually surprised seeing data sharing being so high up because I know of efforts to improve it, but maybe it's not been disseminated yet, but for systems structure as was mentioned, it feeds into everything and like if we don't have the right structures, then there's bureaucracy, there's all, yes."*	Animal Health
Q3	Data Sharing Surprise & System Structure emerges as the core challenge	*"For me, coming from Ghana Health Service, data sharing, yes, it's good when there is an event, let's say an outbreak or there's an emergency, but routinely it's a problem. From animal health and Ghana health service, we have our different channels of reporting so I mean, it's parallel. Everybody's reporting, sometimes there are information that is relevant to both groups, but because we are all following our channels, we are not able to know so that we can respond. So for me the structure is good, every other thing will follow. So what is here, it's OK, the structure is next."*	Human Health
Q4	System Structure emerges as the core challenge	*"No. I'll go for system structure […] You can see that that is the overarching thing that drives all the things here. And then if there was a proper structure, that data sharing and communication challenges wouldn't exist… you can clearly see who the leader, we will see our lines of collaboration, I think that will be the first thing."*	Human Health
Q5	System Structure emerges as the core challenge	*"Can I come in? I'm in the municipal, even district health management team, I'm not part of it … But It's not supposed to be so. [some mix up in the crowd, participants had to be called to order] Let me land and land properly, when I was in [name of city], I will be specific, in [name of district], I was part of it. But when I came to [name of institution], we were, I was part of the health team. But when I came to [name of district], I will state it, during health meetings, I'm not called. So how do I give the report on the health activities of animals to the person. And you know diseases, most of the… when we are talking about this thing, they say human health, they say they are the front-line people but we are worse of in the sense that most of the diseases, they come from animals and we face them squarely. And when you're doing your management team, you don't call the Vets, so how do I give the report?*	Animal Health

		And when I came to the district initially, I wanted data on dog bite cases, It took me almost 3-6 months before they gave it me. I wrote a letter! These are modalities that's why the Vet people they are trying to revenge. When you need anything from health service, they will say … [laughs]multiple … No, I'm being frank, I'm not hiding my feelings. Because some of my people they want to do it that way, when you need something from the health service, "write to the regional", "do this do this", the same thing we want to do. When you want any information from veterinary, we will ask you to write to the region before we give it to you. So that's the problem we are facing."	
Q6	System Structure emerges as the core challenge	*"So, I would also agree with the system structures. I am agreeing with what Doc said, I think that if the structure is good, all this inadequate knowledge or creating awareness at the community level, policies, legislation, political will, workforce, everything will feed into the structure."*	Human Health
Q7	System Structure emerges as the core challenge	*"I think that from my experience, I'm a [professional cadre], and what I found is that my relations with the veterinary tends to be more of a personal basis. When I first moved to where I was, I never saw the vet. The first time I saw the vet was when there was a rabies case, in fact it was a rabies death. The person went to die in another district, the information came back and then I had to physically go and look for the vet, and we found him, he helped us, he gave us all the response activities and all that. When he went on retirement, I knew he was going on retirement I didn't hear from him again. When the new person came, I didn't see the person, I think it was only when Joannishka came when we went to find the new person. We didn't know when the new person had come and they are very divorced. In my district, the person is divorced from the activities of the assembly, that has been my experience. So if I dont physically see the person, If even the person is sitting here kraa i wouldnt even know them … But usually, I mean with the Agric, the Agric person is part of everything so usually when you want to do things around even zoonosis or one health, the first person that comes to mind is the Agric director, not the Vet; it's not intentional. So I think that whoever your …vet people should come, I said get closer to*	Human Health

		the health people and when you have cases, we'll give you. Me for instance, every month, we compile data; dog bite cases the person say they are not giving you, the person didn't want to give to you, you can just click the DHIMS, and it's there. You don't need any magic, there's no magic involved"	
Q8	System Structure emerges as the core challenge	*"Because of the decentralisation, veterinary units at the municipal level is hanging. So that is why you are not seeing most of them in the assembly meetings. Because formerly, we were under the ministry of Agric, department of Agric. But now it's like we belong to civil service while they belong to local government, Ok. So at the district level, we recognise the department of Agric under them so it's only in some few assemblies, they'll write to the vet to be part. So it's not intentional that the person is not coming. That is why we are all trying to say that the system structure is something that is very key, if we have the structure in place, you don't need to tell the person to come. The person knows that automatically, the Agric director is not supposed to come, you know that it's supposed to be a vet. So if the vet is not there, then there should be a vacuum, and then we come and fill in, right. So I think that's what I want to correct for now, later on I'll come back. For example, you see Ghana Health Service, so they report into the DHIMS right? Now the issue is that the district level, there's a whole lot of bureaucracies. We also report through probably our mobile apps or other formats. So even sometimes, they will have a data on dog bites, we'll have data on dog bites, so at the end of the day, we don't even add ours to theirs. OK, so they only get it from the health facilities. But probably, we will also have our data. So that's why we are saying that it's system structure; so at the end the day if they know that well at the end of the month, probably they will call us to probably get what we have that they don't have to add up or something. You understand? So those things are not really there, so everybody is moving on his own lane, but sometimes getting it becomes a challenge."*	Animal Health
Q9	System Structure emerges as the core challenge	*"Doc, I appreciate where you are coming from. The setting may probably vary from district to district, but we have a composition of our district health management team which doesn't include the veterinary officers. However, we have a public health*	Human Health

		Emergency Management Committee, which includes the Veterinary and Health Service. So if you are part of the team and you should be part of the team, you are mandated to have a meeting every quarter that is chaired by the municipal chief executive. Sometimes it doesn't happen because of sometimes lack of political will, no funding for the meeting, but we as health are trying, it's not a measure for the assembly, what is of a measure for the assembly is the health committee, not the public health emergency management committee. So for them they want to have their health committee meetings, which you may probably not be part, but the health sector, which is the Ghana Health service through the municipal health, directorate tries to push for the regular organisation of the Public Health Management committees on a quarterly basis and the issues that come in are funding for the meetings and which doesn't come in very regularly and the lack of political will on the part of the leadership at the municipal assembly level. I think and there's been a. Lot of improvements since COVID came. Initially it wasn't there, but when COVID came, almost all the public health emergency management committee were inaugurated, those that were not inaugurated were inaugurated, and they were meeting quite regularly. It's come down a little bit because COVID has gone down. But still we are still having the Emergency Management Committee meetings. So I think that now we are brothers and sisters, we are collaborating more, but we should look at areas of collaborating better. So for example, when you mentioned the communication and having a platform for communication at the national level, maybe that can also be equally translated to the regional level and the district level where information sharing becomes a little or a bit more easier, yeah."	
Q10	Reintroducing Financial Challenges, but System Structure still stands	*"No. I think if systems are in place, we can find or we can have a way of getting the finances […], yes, if systems are in place and there is a good collaboration, and there's a good leadership, wherever the money is, it will come"*	Human Health
Q11	Reintroducing Financial Challenges, but System Structure still stands	*"Currently in the world, I think there's something that is catching fire; systems, is it systems analysis or… systems approach. If we look at the systems approach, policy falls under it. Leadership, funding and everything comes under it so just like she said,*	Human Health

		if you have your systems and your structure right, everything goes on well. That is why if you look at the yet to be passed Ghana one health policy, the systems approach are only used, all sectors are captured there to the extent that we may not need a particular funding basket for one health activities, but then Veterinary Service will still use the normal funding that they get, but through their activities towards the one health approach or concept. Wildlife same, human health same, they will still use the existing funding that they have to carry out the normal activities that is the activities that they are still carrying; but then the One Health […] yeah both here from the district to the regional to the national level. So systems definitely is number one."	
Q12	Reintroducing Financial Challenges, but System Structure still stands	*"We can build the systems with the support of the finances, so if we don't have the financial support, we cannot build a good system. So I think both of them play a role in making sure that we have the good One Health approach. For instance, if you want to build a surveillance system from top to down, we need some what? Structures; and it is the financial aspect that will help us build those structures in terms of human resources, in terms of infrastructure and in terms of even capacity building. So looking at it in any aspect you want to look at it, I think they are interconnected and all this financial commitment and system structures are also being managed by the political will. We can have all the money [yes][multiple], but if the leadership is not willing to fund it, it's not going to work. Because we can have the policies, the legislation and everything, but once the political head is not interested, it means it won't work."*	Human Health
Q13	Reintroducing Financial Challenges, but System Structure still stands	*"So, I have a contrary view to what he is saying, I think that currently, a lot of the work is already being done. We are all doing it, I don't think that if we are going to do One Health approach, we are going to do some strange new things. It's just that we have to find a way to sync everything and we dont need large baskets of money to do that. We just have to agree that for instance, every month, maybe Ghana Health service will take their IDSR report and leave us a copy, vet will also give their monthly report to Ghana… and it's a structure; so every month at the final level we are doing it, for every created structures of reporting. Some of these don't need*	Human Health

		money to necessarily to do. So I think that, I understand what you were saying; money helps, but One Health from where I sit, is not a strange thing. We already doing it in different, different ways, we just have to find a way to put it all together for it to come together as a one unit so I still stand by my structure…"	
Q14	Reintroducing Financial Challenges, but System Structure still stands	*"Yeah. Yes, I think depending on where you stand, maybe the problem looks different. OK, so I think that in certain groups, maybe money may not seem like an issue, but in certain places, for instance, I don't even have a personal computer. I don't have a structure in place to get a personal computer. How can I keep? I'm in the vet sector, so how do I keep doing reports? For One Health to work, each party has to be equally equipped to play its role. OK, so if you have one part that is recognised, that is equipped but the other part is left behind, we can't do One Health. When I get a rabies case, I can't follow up, I can't even go to where the dog is, I can't go and effect control measures; I can't find out the status of the dog so we can say we'll share reports and things like that, but effectively One Health will not work unless we address the underlying issues. So I'll also just add that the financial, I see it as more of a symptom, OK. But the real cause is with system structure, I also think it's also linked with the political will, thank you"*	Animal Health
Q15	Reintroducing Financial Challenges, but System Structure still stands	*"Just to add something brief to what my senior colleague said. So, finances yes is good, you can have all the money but if things are not in place, you will just displace it. And like Doc said, we already have the systems running, it's for us to probably incorporate it or integrate the whole system. And in fact, the whole thing is that people are not really aware when you go to the district and community level. OK, fine, that's awareness but of course, all these are connected […], but we are trying to say that most of these things, national level, maybe regional level, we talk about it but when it comes to application then it becomes a problem. So like you are saying, let's take the COVID thing for example, when COVID came, at least from the district level we were doing a bit, contact tracing, laboratory level, vets were helping with the confirmation and all that OK. So for the structures we have some in place, but we need to make sure that these things are formalised, you understand? Because if you don't do that, you will always talk about it then getting it from the district level where*	Animal Health

		the activity takes place, it doesn't go on… So we don't need so much money to do these things, it's about how we can use what we have. Of course, going forward, we need more capacity, logistics like Dr [name of AH actor] said, yes."	
Q16	Policy and Leadership Embedded in System Structure	*"Yes, so when we talk about system structure, I think there it is also determined by policy and legislation. But we've left it on one side […].Yes, yes. But I mean we said that apart from the financial challenge, system structure but I think that legislation and policies, it actually guides almost everything. So if we leave it out on the picture; it guides finances, it guides system structure, it guides leadership and collaboration. So if we take it out of the picture, I for one… yeah, yes because without that, all these things will probably not even work, yeah."*	Human Health
Q17	Policy and Leadership Embedded in System Structure	*"I think the systems structure, you know, should be married to the policies and the legalisation. Yes, they mean that we have the system, but they are in silos as they are saying. So if we have a legal framework that will at least facilitate the fact that all the systems in silos will be synchronised, yes, then we are good to go. So this one, the policies and then the system structure will be combined as a single thing, yeah."*	Wildlife Health
Q18	Policy and Leadership Embedded in System Structure	*"Policies … I'm telling you without these things, it won't work. We can have all the structures but the policy will help for it to be implemented."*	Animal Health
Q19	Policy and Leadership Embedded in System Structure	*"Talking about the policies, are the policies there and they are not being implemented? Or they are not there at all, that is the question. […] The reason why I am saying proper policy; when you want to do policy, you need to meet those involved in it, whereby you take haphazard policy and later you realise that it's a wrong policy. That's why I'm talking about the proper one, I know what angle I'm coming from. I know the angle I'm coming from."*	Animal Health

Q20	Existing Policies vs Practice	*I think that both teams have come up with very tangible suggestions or recommendation on how to improve the system, especially with the challenges that we all recognised. Just to add on what they said on bringing the stakeholders on board particularly the veterinary service to the municipal health management committee, that one is a law. It's a policy, the members are already stated in the policy so if you want any additions, then you have to get that legislation legally changed ".”*	Human Health
Q21	Existing Policies vs Practice	*No, no you can actually co-opt members because at that meeting they can agree to co-opt. Because in my district, we have co-opted some people to join the committee. ...yes we have the law, the law has the people but if you think people are necessary to the addition of the committee, you can co-opt. But that one, your Municipal Chief Director must be interested and know why you want to co-opt because it also comes with a financial cost to them.”(*	Human Health
Q22	Workforce Gaps underemphasized	*“did any of the groups look at workforce? I am talking in terms of veterinary. One veterinary officer is for [name of District A] and [name of district B]. It's a challenge, you are looking for Dr [name of vet] and he's at [District B].”*	Human Health
Q23	Workforce Gaps underemphasized	*“My fellow, my fellow veterinarians, I was very disappointed at the inadequate workforce. You know that Wildlife contributes a lot in terms of reporting diseases that are zoonotic in nature. In the whole country, we are just three wildlife vets, ok. This is absolutely in the whole country. We are just three wildlife vets that have to make sure that they report things, to improve disease surveillance of wildlife nature. You can see my face, I already look old … [everyone laughs]. We are jumping from the North to the South, East to West just from here I'm going to the [name of place], there's no rest. So we are asking for collaboration from fellow vets so that we can always tell disease events that are of wildlife nature. And I was thinking that we were going to make sure that this thing [workforce gaps] stood out […]”*	Wildlife Health

NB: All identifying names, locations or professional cadres have been removed. Some quotes reference the first author by name. These references have been retained to preserve narrative authenticity and reflect the researcher's embedded role in the study context
